# The use of the PARIHS framework in implementation research and practice—a citation analysis of the literature

**DOI:** 10.1186/s13012-020-01003-0

**Published:** 2020-08-27

**Authors:** Anna Bergström, Anna Ehrenberg, Ann Catrine Eldh, Ian D. Graham, Kazuko Gustafsson, Gillian Harvey, Sarah Hunter, Alison Kitson, Jo Rycroft-Malone, Lars Wallin

**Affiliations:** 1 Department of Women’s and Children’s health, Uppsala Global Health Research on Implementation and Sustainability (UGHRIS), Uppsala, Sweden; 2 Institute for Global Health, University College London, London, UK; 3 School of Education, Health, and Social Studies, Dalarna University, Falun, Sweden; 4 Adelaide Nursing School, University of Adelaide, Adelaide, Australia; 5 Department of Medicine and Health, Linköping University, Linköping, Sweden; 6 Department of Public Health and Caring Science, Uppsala University, Uppsala, Sweden; 7 School of Epidemiology and Public Health, University of Ottawa, Ottawa, Canada; 8 Ottawa Hospital Research Institute, Ottawa, Canada; 9 University Library, Uppsala University, Uppsala, Sweden; 10 Caring Futures Institute, College of Nursing and Health Sciences, Flinders University, Adelaide, Australia; 11 Green Templeton College, University of Oxford, Oxford, UK; 12 Division of Health Research, Faculty of Health and Medicine, Lancaster University, Lancashire, UK; 13 Department of Health and Care Sciences, The Sahlgrenska Academy, University of Gothenburg, Gothenburg, Sweden

**Keywords:** Citation analysis, PARIHS framework, Implementation science, Knowledge translation

## Abstract

**Background:**

The Promoting Action on Research Implementation in Health Services (PARIHS) framework was developed two decades ago and conceptualizes successful implementation (SI) as a function (f) of the evidence (E) nature and type, context (C) quality, and the facilitation (F), [SI = f (E,C,F)]. Despite a growing number of citations of theoretical frameworks including PARIHS, details of how theoretical frameworks are used remains largely unknown. This review aimed to enhance the understanding of the breadth and depth of the use of the PARIHS framework.

**Methods:**

This citation analysis commenced from four core articles representing the key stages of the framework’s development. The citation search was performed in Web of Science and Scopus. After exclusion, we undertook an initial assessment aimed to identify articles using PARIHS and not only referencing any of the core articles. To assess this, all articles were read in full. Further data extraction included capturing information about where (country/countries and setting/s) PARIHS had been used, as well as categorizing how the framework was applied. Also, strengths and weaknesses, as well as efforts to validate the framework, were explored in detail.

**Results:**

The citation search yielded 1613 articles. After applying exclusion criteria, 1475 articles were read in full, and the initial assessment yielded a total of 367 articles reported to have used the PARIHS framework. These articles were included for data extraction. The framework had been used in a variety of settings and in both high-, middle-, and low-income countries. With regard to types of use, 32% used PARIHS in planning and delivering an intervention, 50% in data analysis, 55% in the evaluation of study findings, and/or 37% in any other way. Further analysis showed that its actual application was frequently partial and generally not well elaborated.

**Conclusions:**

In line with previous citation analysis of the use of theoretical frameworks in implementation science, we also found a rather superficial description of the use of PARIHS. Thus, we propose the development and adoption of reporting guidelines on how framework(s) are used in implementation studies, with the expectation that this will enhance the maturity of implementation science.

Contributions to the literature • Describes how a well-established theoretical framework—PARIHS—has been operationalized in the scientific literature and provides examples of its use in implementation studies.

• The findings underline that descriptions of the use of the framework generally were not that transparent and often partial.

• Findings also point at difficulties in using the framework, such as lack of guidance on key steps to overcome barriers and support implementation

• Identifies the need of common guidelines on how theories, models, and frameworks should be reported in research articles.

## Introduction

There has been an increased use of theoretical frameworks in the field of implementation science in the last decade, with most developed in the last two decades [1, 2]. Tabak et al. identified 61 theoretical models used in dissemination and implementation science [3]. However, while theoretical frameworks are increasingly being cited, more research is needed to understand how they are chosen and applied, and how their use relates to improved implementation outcomes [1, 4]. Variously described in the form of theories, frameworks, or models, all strive to provide conceptual clarity on different aspects of implementation practice and research. For consistency, we will refer to these as theoretical frameworks, or simply “frameworks.”

The Promoting Action on Research Implementation in Health Services (PARIHS) framework is a multi-dimensional framework which was developed to explicitly challenge the pipeline conceptualization of implementation [5]. The PARIHS framework is a commonly used conceptual framework [1, 4] that posits successful implementation (SI) as a function (f) of the nature and type of evidence (E) (including research, clinical experience, patient experience, and local information), the qualities of the context (C) of implementation (including culture, leadership, and evaluation), and the way the implementation process is facilitated (F) (internal and/or external person acting as a facilitator to enable the process of implementation); SI = f(E,C,F). The framework was informed by Rogers’ Diffusion of Innovations [6] and various organizational theories and theories from social science [7] and generated inductively by working with clinical staff to help them understand the practical nature of getting evidence into practice. The PARIHS framework was initially published in 1998 [5] and updated based on a conceptual analysis in 2002 [8] and further primary research [9]. A further refinement was undertaken in 2015 [10], resulting in the integrated or i-PARIHS. Articles using the revised version are not included in the citation analysis reported here. The PARIHS framework has been described as a determinant framework in that it specifies determinants that act as barriers and enablers influencing implementation outcomes [2]. Skolarus et al. [1] identified Kitson et al. [5] as one of the two primary originating sources of influence in their citation analysis of dissemination and implementation frameworks.

Despite the growing number of citations of theoretical frameworks in scientific articles, the detail of how frameworks are used remains largely unknown. Systematic reviews of the application of two other commonly used frameworks [1], the Knowledge to Action framework [11] and the Consolidated Framework for Implementation Research [12], both reported that use of these frameworks, beyond simply citation, was uncommon. While PARIHS has been widely cited, it has also been scrutinized; in 2010, Helfrich et al. published a qualitative critical synthesis of studies that had used the PARIHS framework [13], finding six core concept articles and 18 empirical articles. One of the reported findings was that PARIHS was generally used as an organizing framework for analysis. At the time, no studies used PARIHS prospectively to design implementation strategies [13]. A systematic review applying citation analysis to map the use of PARIHS (similar to those undertaken for the Knowledge to Action framework (KTA) [11] and the Consolidated Framework for Implementation Research (CFIR) [12]) has not yet been performed.

Systematic reviews can contribute to the development of existing theoretical frameworks by critically reviewing what authors state as their weaknesses and strengths; they can also direct future and current users of frameworks to examples of using the frameworks in different ways. To contribute to this development from the perspective of the PARIHS framework, we undertook a citation analysis of the published peer-reviewed literature that focused on the reported use of PARIHS (and its main elements), in what contexts the framework has been applied, and what scholars who have used the PARIHS framework (and its main elements) report as its strengths, limitations, and validity.

## Methods

The method used for this study is citation analysis, i.e., the examination of the frequency and patterns of citations in scientific articles, in this case articles citing the core PARIHS framework publications. A team of researchers with engagement in the development and/or use of the PARIHS framework was constituted. Initially, the group decided on the core publications for the citation analysis. Four articles were selected as they represented the key stages of the framework’s development, namely the original paper that described PARIHS, plus three subsequent papers that informed and outlined revisions to the framework:

1. Kitson A, Harvey G, McCormack B. Enabling the implementation of evidence-based practice: a conceptual framework. Qual Health Care. 1998;7(3):149-58.
2. Rycroft-Malone J, Kitson A, Harvey G, McCormack B, Seers K, Titchen A, et al. Ingredients for change: revisiting a conceptual framework. BMJ Quality Saf. 2002;11(2):174-80.
3. Rycroft-Malone J, Harvey G, Seers K, Kitson A, McCormack B, Titchen A. An exploration of the factors that influence the implementation of evidence into practice. J Clin Nurs. 2004;13(8):913-24.
4. Kitson AL, Rycroft-Malone J, Harvey G, McCormack B, Seers K, Titchen A. Evaluating the successful implementation of evidence into practice using the PARiHS framework: theoretical and practical challenges. Implement Sci. 2008;3:1.

### Citation search

Citation searches were performed by an information specialist (KG) to retrieve published articles citing any of the four core articles. The searches were performed in two citation databases: Web of Science and Scopus. The first searches were performed between 31 March 2016 and 1 April 2016. Later, 6 September 2019, additional searches were performed in respective databases. These searches were limited to citations that were published 1 April 2016–31 August 2019 to update the result from the first searches. All citations that were published September 1998 (i.e., when Kitson et al 1998 was published)–31 August 2019 (i.e., prior to the search date) in respective databases were collected in EndNote Library. Endnote was used for checking duplicates and retrieving full texts. To manage the scope of the citation analysis, we opted to only include articles in English published in peer-reviewed scientific journals. The searches in Web of Science were, because of the subscription, limited to Web of Science Core Collection without Book Citation Index.

### Data extraction

**Fig. 1:**
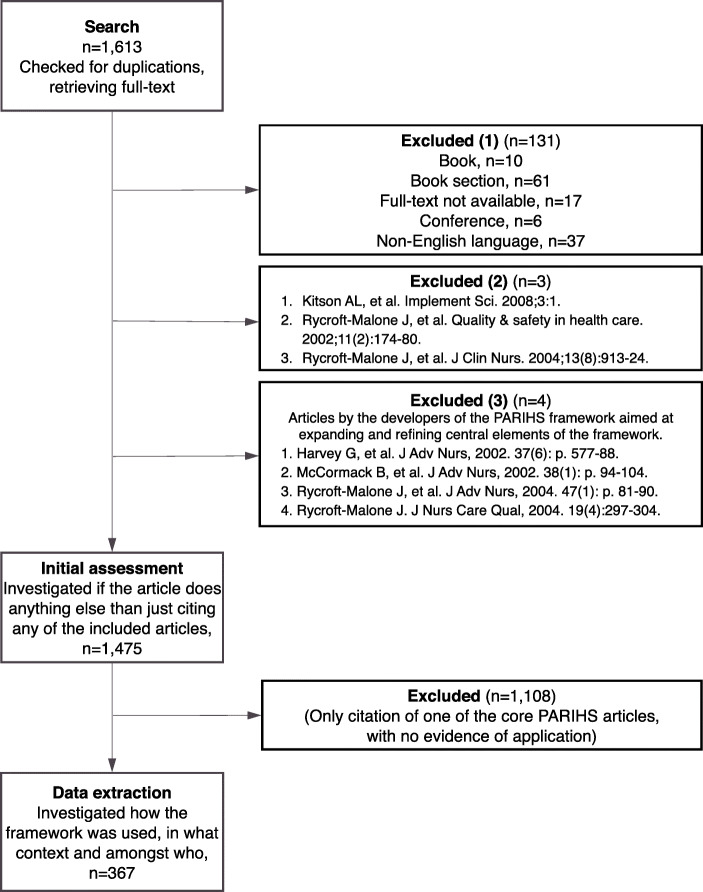
Adapted PRISMA flow diagram

The Preferred Reporting Items for Systematic Reviews and Meta-Analyses (PRISMA) flow diagram [14] for the data extraction is provided in Fig. 1. Initially, an assessment to identify the articles that used the PARIHS framework in any other way than merely referencing one or more of the core articles was performed (Additional file 1). For this initial assessment, all articles were read in full. After identifying articles where the PARIHS framework was used, data extraction was undertaken using a tailor-made data capture form (Additional file 1). The data capture form was developed and piloted in iterative cycles by the research team. Apart from capturing information about where (country/countries and setting/s) and with whom (professional groups and roles) PARIHS had been applied, the form included questions on whether PARIHS was used in one or more of the following ways:

1. In planning and delivering an intervention,
2. In data analysis,
3. In the evaluation of study findings, and/or
4. In any other way.

Each of these questions was followed by an open-ended item for extracting information on how this was reported [15]. To enhance reliability and data richness, each reviewer copy-pasted sections of the article corresponding to the open-ended reply into the data extraction form when appropriate and indicated page, column, and row. Two additional items captured whether the PARIHS framework had been tested or validated, as well as any reported strengths and weaknesses of the framework. Thus, we report on what the authors of the included articles claim to have done, rather than a judgment as to how and to what extent they actually used the PARIHS framework.

For data extraction and validation, the research team was divided into four pairs, ensuring that each article was assessed separately by at least two research team members. The pairs received batches of 20 articles at a time. Variations in the assessments were discussed until consensus was reached within the pair(s). Further, queries detected within the pairs were raised and discussed with the whole research team, until consensus was achieved. Regular whole-team online meetings were held to consolidate findings between every new batch of articles and throughout the development and analysis process. In total, the group had > 20 online meetings and four face-to-face meetings from the initial establishment of the group in January 2015.

### Data analysis

Categorical data were analyzed using descriptive statistics, whereas the open-ended items were analyzed qualitatively [16], including the collated extractions of data to illustrate each of the four types of use (i.e., how the PARIHS framework was depicted in terms of (1) planning and delivering an intervention, (2) analysis, (3) evaluation of study findings, and/or (4) in any other way).

Applying a content analysis approach [17], members of the research team worked separately with the texts extracted from the reviewed articles. The extracts for each open-ended item were read and reread, to get a sense of the whole. Next, variations were identified and formed as categories. Findings for each question were summarized in short textual descriptions, which were shared with the whole team. In a face-to-face meeting, the data relating to each question were critically discussed and comparisons were made between the findings for each question, to identify overlaps and relationships about how PARIHS has been used.

## Results

After duplicate control, 1613 references remained. These were sorted by language and type of publication. In this phase, 131 references categorized as books, book chapters, conference proceedings, and publications written in non-English language were excluded. Also, three of the four core articles (i.e., the three citing Kitson et al. [5] which was the starting point for development of the PARIHS framework and therefore did not appear in the citation search) were excluded from the database [8, 9, 18], as were four articles expanding and refining PARIHS [19–22]. Accordingly, 1475 articles remained, and after the assessment excluding those merely citing PARIHS, a further 1108 articles were excluded, leaving 367 articles that cited one or more of the core articles, and made explicit use of the PARIHS framework (see Fig. 1 and Table 1).

**Table 1:** List of articles for data extraction for citation analysis of the use of the PARIHS framework. The table is sorted on type of article, type of use of the PARIHS framework, author and year of publication

Authors	Ref	Year of publ.	Full title	Country(ies)	Setting	PARIHS used:				Type of article
						To plan/deliver an intervention	In the analysis^**a**^	In the evaluation of findings	In any other way	
Chinman, Daniels, et al.	[23]	2017	Provision of peer specialist services in VA patient aligned care teams: Protocol for testing a cluster randomized implementation trial	USA	Primary health care setting	✓	✓		✓	Protocol
Gordon, Lee, et al.	[24]	2018	A complex culturally targeted intervention to reduce Hispanic disparities in living kidney donor transplantation: An effectiveness-implementation hybrid study protocol	USA	Community/Social care setting	✓	✓		✓	Protocol
Roberge, Fournier, et al.	[25]	2013	Implementing a knowledge application program for anxiety and depression in community-based primary mental health care: A multiple case study research protocol	Canada	Primary health care setting	✓	✓		✓	Protocol
Blanco-Mavillard, Bennasar-Veny, et al.	[26]	2018	Implementation of a knowledge mobilization model to prevent peripheral venous catheter-related adverse events: PREBACP study-a multicenter cluster-randomized trial protocol	Spain	Hospital setting	✓	✓			Protocol
Bucknall, Harvey, et al.	[27]	2017	Prioritizing Responses Of Nurses To deteriorating patient Observations (PRONTO) protocol: Testing the effectiveness of a facilitation intervention in a pragmatic, cluster-randomized trial with an embedded process evaluation and cost analysis	Australia	Hospital setting	✓	✓			Protocol
Chouinard, Hudon, et al.	[28]	2013	Case management and self-management support for frequent users with chronic disease in primary care: A pragmatic randomized controlled trial	Canada	Primary health care setting	✓	✓			Protocol
Cully, Armento, et al.	[29]	2012	Brief cognitive behavioral therapy in primary care: a hybrid type 2 patient-randomized effectiveness-implementation design	USA	Primary health care setting	✓	✓			Protocol
Gurung, Jha, et al.	[30]	2019	Scaling Up Safer Birth Bundle Through Quality Improvement in Nepal (SUSTAIN) a stepped wedge cluster randomized controlled trial in public hospitals	Nepal	Hospital setting	✓	✓			Protocol
Owen, Drummond, et al.	[31]	2013	Monitoring and managing metabolic effects of antipsychotics: A cluster randomized trial of an intervention combining evidence-based quality improvement and external facilitation	USA	Multiple settings	✓	✓			Protocol
Powell, Kitson, et al.	[32]	2013	A study protocol for applying the co-creating knowledge translation framework to a population health study	Australia	Public health	✓	✓			Protocol
Rycroft-Malone, Anderson, et al.	[33]	2014	Accessibility and implementation in UK services of an effective depression relapse prevention program - mindfulness-based cognitive therapy (MBCT): ASPIRE study protocol	UK	Not reported	✓	✓			Protocol
Rycroft-Malone, Dopson, et al.	[34]	2009	Study protocol for the translating research in elder care (TREC): Building context through case studies in long-term care project (project two)	Canada	Community/Social care setting	✓	✓			Protocol
Rycroft-Malone, Wilkinson, et al.	[35]	2011	Implementing health research through academic and clinical partnerships: A realistic evaluation of the Collaborations for Leadership in Applied Health Research and Care (CLAHRC)	UK	Multiple settings	✓	✓			Protocol
Kilbourne, Almirall, et al.	[36]	2014	Protocol: Adaptive Implementation of Effective Programs Trial (ADEPT): Cluster randomized SMART trial comparing a standard versus enhanced implementation strategy to improve outcomes of a mood disorders program	USA	Community/Social care setting	✓			✓	Protocol
McGilton, Davis, et al.	[37]	2012	An inpatient rehabilitation model of care targeting patients with cognitive impairment	Canada	Multiple settings	✓			✓	Protocol
Botti, Kent, et al.	[38]	2014	Development of a Management Algorithm for Post-operative Pain (MAPP) after total knee and total hip replacement: Study rationale and design	Australia	Hospital setting	✓				Protocol
Cadilhac, Andrew, et al.	[39]	2018	Improving quality and outcomes of stroke care in hospitals: Protocol and statistical analysis plan for the Stroke123 implementation study	Australia	Hospital setting	✓				Protocol
Perez, Russo, et al.	[40]	2013	Comparison of high and low intensity contact between secondary and primary care to detect people at ultra-high risk for psychosis: Study protocol for a theory-based, cluster randomized controlled trial	UK	Primary health care setting	✓				Protocol
Ray-Barruel, Cooke, et al.	[41]	2018	Implementing the I-DECIDED clinical decision-making tool for peripheral intravenous catheter assessment and safe removal: protocol for an interrupted time-series study	Australia	Hospital setting	✓				Protocol
Saint, Olmsted, et al.	[42]	2009	Translating health care-associated urinary tract infection prevention research into practice via the bladder bundle	USA	Hospital setting	✓				Protocol
Sampson, Feast, et al.	[43]	2019	Evidence-based intervention to reduce avoidable hospital admissions in care home residents (the Better Health in Residents in Care Homes (BHiRCH) study): Protocol for a pilot cluster randomized trial	UK	Community/Social care setting	✓				Protocol
Seers, Cox, et al.	[44]	2012	FIRE (facilitating implementation of research evidence): A study protocol	UK, Ireland, Sweden, Netherlands	Community/Social care setting	✓				Protocol
Skene, Gerrish, et al.	[45]	2016	Developing family-centered care in a neonatal intensive care unit: An action research study protocol	UK	Hospital setting	✓				Protocol
Wallin, Målqvist, et al.	[46]	2011	Implementing knowledge into practice for improved neonatal survival; A cluster-randomized, community-based trial in Quang Ninh province, Vietnam	Vietnam	Community/Social care setting	✓				Protocol
Conklin, Kothari, et al.	[47]	2011	Knowledge-to-action processes in SHRTN collaborative communities of practice: A study protocol	Canada	Multiple settings		✓		✓	Protocol
Estabrooks, Squires, et al.	[48]	2009	Study protocol for the translating research in elder care (TREC): Building context - An organizational monitoring program in long-term care project (project one)	Canada	Community/Social care setting		✓		✓	Protocol
Kitson, Schultz, et al.	[49]	2013	The prevention and reduction of weight loss in an acute tertiary care setting: Protocol for a pragmatic stepped wedge randomized cluster trial (the PRoWL project)	Australia	Hospital setting		✓		✓	Protocol
Noyes, Williams, et al.	[50]	2010	Evidence into practice: Evaluating a child-centred intervention for diabetes medicine management The EPIC Project	UK	Multiple settings		✓		✓	Protocol
Chao, Chang, et al.	[51]	2016	Adjunctive acupuncture for pain and symptom management in the inpatient setting: protocol for a pilot hybrid effectiveness-implementation study	USA	Hospital setting		✓			Protocol
Hack, Ruether, et al.	[52]	2011	Study protocol: Addressing evidence and context to facilitate transfer and uptake of consultation recording use in oncology: A knowledge translation implementation study	Canada	Hospital setting		✓			Protocol
Stetler, Ritchie, et al.	[53]	2007	Improving quality of care through routine, successful implementation of evidence-based practice at the bedside: An organizational case study protocol using the Pettigrew and Whipp model of strategic change	USA	Hospital setting		✓			Protocol
Urquhart, Porter, et al.	[54]	2012	Exploring the interpersonal-, organization-, and system-level factors that influence the implementation and use of an innovation-synoptic reporting-in cancer care	Canada	Hospital setting		✓			Protocol
Watkins, Nagle, et al.	[55]	2017	Labouring Together: Collaborative alliances in maternity care in Victoria, Australia-protocol of a mixed-methods study	Australia	Hospital setting		✓			Protocol
De Pedro-Gómez, Morales-Asencio, et al.	[56]	2012	Determining factors in evidence-based clinical practice among hospital and primary care nursing staff	Spain	Multiple settings				✓	Protocol
Slaughter, Estabrooks, et al.	[57]	2013	Sustaining Transfers through Affordable Research Translation (START): Study protocol to assess knowledge translation interventions in continuing care settings	Canada	Community/Social care setting				✓	Protocol
Eriksson, Huy, et al.	[58]	2016	Process evaluation of a knowledge translation intervention using facilitation of local stakeholder groups to improve neonatal survival in the Quang Ninh province, Vietnam	Vietnam	Primary health care setting	✓	✓	✓	✓	Empirical study
Eriksson, Nga, et al.	[59]	2011	Newborn care and knowledge translation - perceptions among primary healthcare staff in northern Vietnam	Vietnam	Community/Social care setting	✓	✓	✓	✓	Empirical study
Long-Tounsel, Wilson, et al.	[60]	2014	Urban and Suburban Hospital System Implementation of Multipoint Access Targeted Temperature Management in Postcardiac Arrest Patients	USA	Hospital setting	✓	✓	✓	✓	Empirical study
McWilliam, Kothari, et al.	[61]	2009	Evolving the theory and praxis of knowledge translation through social interaction: A social phenomenological study	Canada	Community/Social care setting	✓	✓	✓	✓	Empirical study
Obrecht, Van Hulle Vincent, et al.	[62]	2014	Implementation of evidence-based practice for a pediatric pain assessment instrument	USA	Hospital setting	✓	✓	✓	✓	Empirical study
Allen, Hall, et al.	[63]	2018	Improving hospital environmental hygiene with the use of a targeted multi-modal bundle strategy	Australia	Hospital setting	✓	✓	✓		Empirical study
Bahtsevani and Idvall	[64]	2016	To Assess Prerequisites before an Implementation Strategy in an Orthopaedic Department in Sweden	Sweden	Hospital setting	✓	✓	✓		Empirical study
Bamford, Rothwell, et al.	[65]	2013	Improving care for people after stroke: How change was actively facilitated	UK	Multiple settings	✓	✓	✓		Empirical study
Brenner, Breshears, et al.	[66]	2011	Implementation of a Suicide Nomenclature within Two VA Healthcare Settings	USA	Multiple settings	✓	✓	✓		Empirical study
Brown and McCormack	[67]	2016	Exploring psychological safety as a component of facilitation within the Promoting Action on Research Implementation in Health Services framework	UK	Hospital setting	✓	✓	✓		Empirical study
Diffin, Ewing, et al.	[68]	2018	Facilitating successful implementation of a person-centred intervention to support family carers within palliative care: a qualitative study of the Carer Support Needs Assessment Tool (CSNAT) intervention	UK	Multiple settings	✓	✓	✓		Empirical study
Diffin, Ewing, et al.	[69]	2018	The Influence of Context and Practitioner Attitudes on Implementation of Person-Centered Assessment and Support for Family Carers Within Palliative Care	UK	Community/Social care setting	✓	✓	✓		Empirical study
Drainoni, Koppelman, et al.	[70]	2016	Why is it so hard to implement change? A qualitative examination of barriers and facilitators to distribution of naloxone for overdose prevention in a safety net environment	USA	Hospital setting	✓	✓	✓		Empirical study
Ellis, Howard, et al.	[71]	2005	From workshop to work practice: An exploration of context and facilitation in the development of evidence-based practice	Australia	Hospital setting	✓	✓	✓		Empirical study
Gerrish, Laker, et al.	[72]	2016	Enhancing the quality of oral nutrition support for hospitalized patients: a mixed methods knowledge translation study (The EQONS study)	UK	Hospital setting	✓	✓	✓		Empirical study
Gesthalter, Koppelman, et al.	[73]	2017	Evaluations of Implementation at Early-Adopting Lung Cancer Screening Programs: Lessons Learned	USA	Multiple settings	✓	✓	✓		Empirical study
Harris, Jones, et al.	[74]	2015	Changing practice to support self-management and recovery in mental illness: Application of an implementation model	Australia	Community/Social care setting	✓	✓	✓		Empirical study
Harvey, McCormack, et al.	[75]	2018	Designing and implementing two facilitation interventions within the "Facilitating Implementation of Research Evidence (FIRE)' study: a qualitative analysis from an external facilitators' perspective	UK, Ireland, Netherlands, Sweden	Community/Social care setting	✓	✓	✓		Empirical study
Houle, Charrois, et al.	[76]	2017	A randomized controlled study of practice facilitation to improve the provision of medication management services in Alberta community pharmacies	Canada	Community/Social care setting	✓	✓	✓		Empirical study
Jangland and Gunningberg	[77]	2017	Improving patient participation in a challenging context: a 2-year evaluation study of an implementation project	Sweden	Hospital setting	✓	✓	✓		Empirical study
Lewis, Kitson, et al.	[78]	2016	Improving oral health for older people in the home care setting: An exploratory implementation study	Australia	Home-based care	✓	✓	✓		Empirical study
Lindsay, Kauth, et al.	[79]	2015	Implementation of Video Telehealth to Improve Access to Evidence-Based Psychotherapy for Posttraumatic Stress Disorder	USA	Multiple settings	✓	✓	✓		Empirical study
Mekki, Øye, et al.	[80]	2017	The inter-play between facilitation and context in the promoting action on research implementation in health services framework: A qualitative exploratory implementation study embedded in a cluster randomized controlled trial to reduce restraint in nursing homes	Norway	Community/Social care setting	✓	✓	✓		Empirical study
Parlour and McCormack	[81]	2012	Blending critical realist and emancipatory practice development methodologies: Making critical realism work in nursing research	Ireland	Community/Social care setting	✓	✓	✓		Empirical study
Persson, Nga, et al.	[82]	2013	Effect of Facilitation of Local Maternal-and-Newborn Stakeholder Groups on Neonatal Mortality: Cluster-Randomized Controlled Trial	Vietnam	Primary health care setting	✓	✓	✓		Empirical study
Rycroft-Malone, Fontenla, et al.	[83]	2009	Protocol-based care: The standardisation of decision-making?	UK	Hospital setting	✓	✓	✓		Empirical study
Rycroft-Malone, Seers, et al.	[84]	2012	A pragmatic cluster randomised trial evaluating three implementation interventions	UK	Hospital setting	✓	✓	✓		Empirical study
Rycroft-Malone, Seers, et al.	[85]	2013	The role of evidence, context, and facilitation in an implementation trial: Implications for the development of the PARIHS framework	UK	Hospital setting	✓	✓	✓		Empirical study
Rycroft-Malone, Seers, et al.	[86]	2018	A realist process evaluation within the Facilitating Implementation of Research Evidence (FIRE) cluster randomised controlled international trial: An exemplar	Ireland, UK, Netherlands, Sweden	Community/Social care setting	✓	✓	✓		Empirical study
Slaughter and Estabrooks	[87]	2013	Optimizing the mobility of residents with dementia: A pilot study promoting healthcare aide uptake of a simple mobility innovation in diverse nursing home settings	Canada	Community/Social care setting	✓	✓	✓		Empirical study
Sving, Fredriksson, et al.	[88]	2017	Getting evidence-based pressure ulcer prevention into practice: a process evaluation of a multifaceted intervention in a hospital setting	Sweden	Hospital setting	✓	✓	✓		Empirical study
Walsh, Ford, et al.	[89]	2017	The Development and Implementation of a Participatory and Solution-Focused Framework for Clinical Research: A case example	Australia	Hospital setting	✓	✓	✓		Empirical study
Mignogna, Hundt, et al.	[90]	2014	Implementing brief cognitive behavioral therapy in primary care: A pilot study	USA	Primary health care setting	✓	✓		✓	Empirical study
Alkema and Frey	[91]	2006	Implications of translating research into practice: A medication management intervention	USA	Home-based care	✓	✓			Empirical study
Kilbourne, Abraham, et al.	[92]	2013	Cluster randomized adaptive implementation trial comparing a standard versus enhanced implementation intervention to improve uptake of an effective re-engagement program for patients with serious mental illness	USA	Multiple settings	✓	✓			Empirical study
Mignogna, Martin, et al.	[93]	2018	I had to somehow still be flexible: exploring adaptations during implementation of brief cognitive behavioral therapy in primary care	USA	Primary health care setting	✓	✓			Empirical study
Westergren	[94]	2012	Action-oriented study circles facilitate efforts in nursing homes to go from feeding to serving: Conceptual perspectives on knowledge translation and workplace learning	Sweden	Community/Social care setting	✓	✓			Empirical study
Baloh, Zhu, et al.	[95]	2018	Types of internal facilitation activities in hospitals implementing evidence-based interventions	USA	Hospital setting	✓		✓	✓	Empirical study
Snelgrove-Clarke, Davies, et al.	[96]	2015	Implementing a Fetal Health Surveillance Guideline in Clinical Practice: A Pragmatic Randomized Controlled Trial of Action Learning	Canada	Hospital setting	✓		✓	✓	Empirical study
Wallin, Rudberg, et al.	[97]	2005	Staff experiences in implementing guidelines for Kangaroo Mother Care - A qualitative study	Sweden	Hospital setting	✓		✓	✓	Empirical study
Bidassie, Williams, et al.	[98]	2015	Key components of external facilitation in an acute stroke quality improvement collaborative in the Veterans Health Administration	USA	Hospital setting	✓		✓		Empirical study
Doran, Haynes, et al.	[99]	2012	The role of organizational context and individual nurse characteristics in explaining variation in use of information technologies in evidence based practice	Canada	Multiple settings	✓		✓		Empirical study
Fortney, Enderle, et al.	[100]	2012	Implementation outcomes of evidence-based quality improvement for depression in VA community based outpatient clinics	USA	Multiple settings	✓		✓		Empirical study
Foss, Kvigne, et al.	[101]	2014	A model (CMBP) for collaboration between university college and nursing practice to promote research utilization in students' clinical placements: A pilot study	Norway	Educational setting	✓		✓		Empirical study
Johnson, Ostaszkiewicz, et al.	[102]	2009	Moving beyond resistance to restraint minimization: A case study of change management in aged care	Australia	Community/Social care setting	✓		✓		Empirical study
Kavanagh, Stevens, et al.	[103]	2010	Process evaluation of appreciative inquiry to translate pain management evidence into pediatric nursing practice	Canada	Hospital setting	✓		✓		Empirical study
Kinley, Stone, et al.	[104]	2014	The effect of using high facilitation when implementing the Gold Standards Framework in Care Homes program: A cluster randomized controlled trial	UK	Community/Social care setting	✓		✓		Empirical study
Lewis, Harvey, et al.	[105]	2019	Can oral healthcare for older people be embedded into routine community aged care practice? A realist evaluation using normalization process theory	Australia	Home-based care	✓		✓		Empirical study
McGilton, Sorin-Peters, et al.	[106]	2018	The effects of an interprofessional patient-centered communication intervention for patients with communication disorders	Canada	Hospital setting	✓		✓		Empirical study
McLean, Torkington, et al.	[107]	2019	Development, Implementation, and Outcomes of Post-stroke Mood Assessment Pathways: Implications for Social Workers	Australia	Hospital setting	✓		✓		Empirical study
O'Halloran, Cran, et al.	[108]	2007	Factors affecting adherence to use of hip protectors among residents of nursing homes - A correlation study	UK	Community/Social care setting	✓		✓		Empirical study
Pallangyo, Mbekenga, et al.	[109]	2017	“If really we are committed things can change, starting from us”: Healthcare providers’ perceptions of postpartum care and its potential for improvement in low-income suburbs in Dar es Salaam, Tanzania	Tanzania	Multiple settings	✓		✓		Empirical study
Pallangyo, Mbekenga, et al.	[110]	2018	Implementation of a facilitation intervention to improve postpartum care in a low-resource suburb of Dar es Salaam, Tanzania	Tanzania	Multiple settings	✓		✓		Empirical study
Russell-Babin and Miley	[111]	2013	Implementing the best available evidence in early delirium identification in elderly hip surgery patients	USA	Hospital setting	✓		✓		Empirical study
Rycroft-Malone, Wilkinson, et al.	[112]	2013	Collaborative action around implementation in Collaborations for Leadership in Applied Health Research and Care: toward a program theory	UK	Multiple settings	✓		✓		Empirical study
Seers, Rycroft-Malone, et al.	[113]	2018	Facilitating Implementation of Research Evidence (FIRE): An international cluster randomized controlled trial to evaluate two models of facilitation informed by the Promoting Action on Research Implementation in Health Services (PARIHS) framework	UK, Ireland, Netherlands, Sweden	Community/Social care setting	✓		✓		Empirical study
Sigel, Kramer, et al.	[114]	2013	Statewide dissemination of trauma-focused cognitive-behavioral therapy (TF-CBT)	USA	Multiple settings	✓		✓		Empirical study
Stevens, Yamada, et al.	[115]	2016	Pain assessment and management after a knowledge translation booster intervention	Canada	Hospital setting	✓		✓		Empirical study
Sving, Högman, et al.	[116]	2016	Getting evidence-based pressure ulcer prevention into practice: a multi-faceted unit-tailored intervention in a hospital setting	Sweden	Hospital setting	✓		✓		Empirical study
Tian, Yang, et al.	[117]	2017	Implementation of evidence into practice for cancer-related fatigue management of hospitalized adult patients using the PARIHS framework	China	Hospital setting	✓		✓		Empirical study
Tucker, Bieber, et al.	[118]	2012	Outcomes and Challenges in Implementing Hourly Rounds to Reduce Falls in Orthopedic Units	USA	Hospital setting	✓		✓		Empirical study
Weir, Brunker, et al.	[119]	2017	Making cognitive decision support work: Facilitating adoption, knowledge and behavior change through QI	USA	Primary health care setting	✓		✓		Empirical study
Williams, Woodby, et al.	[120]	2014	Formative Evaluation of a Multi-Component, Education-Based Intervention to Improve Processes of End-of-Life Care	USA	Hospital setting	✓		✓		Empirical study
Yurumezoglu and Kocaman	[121]	2012	Pilot study for evidence-based nursing management: Improving the levels of job satisfaction, organizational commitment, and intent to leave among nurses in Turkey	Turkey	Hospital setting	✓		✓		Empirical study
Brosey and March	[122]	2015	Effectiveness of Structured Hourly Nurse Rounding on Patient Satisfaction and Clinical Outcomes	USA	Hospital setting	✓			✓	Empirical study
Chinman, Acosta, et al.	[123]	2013	Intervening with Practitioners to Improve the Quality of Prevention: One-Year Findings from a Randomized Trial of Assets-Getting To Outcomes	USA	Community/Social care setting	✓			✓	Empirical study
Glegg	[124]	2010	Knowledge brokering as an intervention in paediatric rehabilitation practice	Canada	Not reported	✓			✓	Empirical study
Harvey, Oliver, et al.	[125]	2015	Improving the identification and management of chronic kidney disease in primary care: Lessons from a staged improvement collaborative	UK	Primary health care setting	✓			✓	Empirical study
Humphreys, Harvey, et al.	[126]	2012	A collaborative project to improve identification and management of patients with chronic kidney disease in a primary care setting in Greater Manchester	UK	Not reported	✓			✓	Empirical study
Kauth, Sullivan, et al.	[127]	2010	Employing external facilitation to implement cognitive behavioral therapy in VA clinics: A pilot study	USA	Not reported	✓			✓	Empirical study
Almblad, Siltberg, et al.	[128]	2018	Implementation of Pediatric Early Warning Score; Adherence to Guidelines and Influence of Context	Sweden	Hospital setting	✓				Empirical study
Amaya-Jackson, Hagele, et al.	[129]	2018	Pilot to policy: statewide dissemination and implementation of evidence-based treatment for traumatized youth	USA	Community/Social care setting	✓				Empirical study
Anderson, Zlateva, et al.	[130]	2016	Improving pain care through implementation of the stepped care model at a multisite community health center	USA	Primary health care setting	✓				Empirical study
Bailey, Williams, et al.	[131]	2014	Intervention to Improve Care at Life's End in Inpatient Settings: The BEACON Trial	USA	Hospital setting	✓				Empirical study
Bunch, Leasure, et al.	[132]	2016	Implementation of a rapid chest pain protocol in the emergency department: A quality improvement project	USA	Hospital setting	✓				Empirical study
Gutmanis, Snyder, et al.	[133]	2015	Health care redesign for responsive behaviours - The behavioural supports Ontario experience: Lessons learned and keys to success	Canada	Multiple settings	✓				Empirical study
McGilton, Rochon, et al.	[134]	2017	Can We Help Care Providers Communicate More Effectively With Persons Having Dementia Living in Long-Term Care Homes?	Canada	Community/Social care setting	✓				Empirical study
Murphy, Gardner, et al.	[135]	2014	A theory-informed approach to mental health care capacity building for pharmacists	Canada	Pharmacies	✓				Empirical study
Musanti, O'Keefe, et al.	[136]	2012	Partners in caring: An innovative nursing model of care delivery	USA	Hospital setting	✓				Empirical study
O'Brien, Redley, et al.	[137]	2018	STOPDVTs: Development and testing of a clinical assessment tool to guide nursing assessment of postoperative patients for Deep Vein Thrombosis	Australia	Hospital setting	✓				Empirical study
Orsted, Rosenthal, et al.	[138]	2009	Pressure ulcer awareness and prevention program: A quality improvement program through the Canadian association of wound care	Canada	Multiple settings	✓				Empirical study
Rutledge and Skelton	[139]	2011	Clinical expert facilitators of evidence-based practice: A community hospital program	USA	Hospital setting	✓				Empirical study
Ryan, Barnett, et al.	[140]	2013	Geriatrics, interprofessional practice, and interorganizational collaboration: A knowledge-to-practice intervention for primary care teams	Canada	Primary health care setting	✓				Empirical study
Sadasivam, Hogan, et al.	[141]	2013	Implementing point of care "e-referrals" in 137 clinics to increase access to a quit smoking internet system: The Quit-Primo and National Dental PBRN HI-QUIT Studies	USA	Primary health care setting	✓				Empirical study
Smith, Almirall, et al.	[142]	2019	Change in Patient Outcomes After Augmenting a Low-level Implementation Strategy in Community Practices That Are Slow to Adopt a Collaborative Chronic Care Model A Cluster Randomized Implementation Trial	USA	Community/Social care setting	✓				Empirical study
Stevens, Yamada, et al.	[143]	2014	Pain in hospitalized children: Effect of a multidimensional knowledge translation strategy on pain process and clinical outcomes	Canada	Hospital setting	✓				Empirical study
Tilson, Mickan, et al.	[144]	2016	Promoting physical therapists' use of research evidence to inform clinical practice: part 3-long term feasibility assessment of the PEAK program	USA	Multiple settings	✓				Empirical study
Tistad, Palmcrantz, et al.	[145]	2016	Developing leadership in managers to facilitate the implementation of national guideline recommendations: A process evaluation of feasibility and usefulness	Sweden	Multiple settings	✓				Empirical study
Toole, Stichler, et al.	[146]	2013	Promoting nurses' knowledge in evidence-based practice: Do educational methods matter?	USA	Hospital setting	✓				Empirical study
Westergren, Axelsson, et al.	[147]	2009	Study circles improve the precision in nutritional care in special accommodations	Sweden	Community/Social care setting	✓				Empirical study
Young, Banks, et al.	[148]	2018	Improving nutrition care and intake for older hospital patients through system-level dietary and mealtime interventions	Australia	Hospital setting	✓				Empirical study
Balbale, Hill, et al.	[149]	2015	Evaluating implementation of methicillin-resistant Staphylococcus aureus (MRSA) prevention guidelines in spinal cord injury centers using the PARIHS framework: A mixed methods study	USA	Hospital setting		✓	✓	✓	Empirical study
Bergström, Peterson, et al.	[150]	2012	Knowledge translation in Uganda: A qualitative study of Ugandan midwives' and managers' perceived relevance of the sub-elements of the context cornerstone in the PARIHS framework	Uganda	Multiple settings		✓	✓	✓	Empirical study
Boblin, Ireland, et al.	[151]	2013	Using Stake's Qualitative Case Study Approach to Explore Implementation of Evidence-Based Practice	Canada	Hospital setting		✓	✓	✓	Empirical study
Cammer, Morgan, et al.	[152]	2014	The hidden complexity of long-term care: How context mediates knowledge translation and use of best practices	Canada	Community/Social care setting		✓	✓	✓	Empirical study
Cummings, Estabrooks, et al.	[153]	2007	Influence of organizational characteristics and context on research utilization	Canada	Hospital setting		✓	✓	✓	Empirical study
Estabrooks, Midodzi, et al.	[154]	2007	Predicting research use in nursing organizations: A multilevel analysis	Canada	Hospital setting		✓	✓	✓	Empirical study
Estabrooks, Squires, et al.	[155]	2009	Development and assessment of the Alberta Context Tool	Canada	Hospital setting		✓	✓	✓	Empirical study
Estabrooks, Squires, et al.	[156]	2011	Advancing the argument for validity of the Alberta Context Tool with healthcare aides in residential long-term care	Canada	Community/Social care setting		✓	✓	✓	Empirical study
Gagliardi, Webster, et al.	[157]	2014	How does context influence collaborative decision-making for health services planning, delivery and evaluation?	Canada	Hospital setting		✓	✓	✓	Empirical study
Gibb	[158]	2013	An environmental scan of an aged care workplace using the PARiHS model: Assessing preparedness for change	Australia	Community/Social care setting		✓	✓	✓	Empirical study
Helfrich, Li, et al.	[159]	2009	Organizational readiness to change assessment (ORCA): Development of an instrument based on the promoting action on research in health services (PARIHS) framework	USA	Multiple settings		✓	✓	✓	Empirical study
Kristensen and Hounsgaard	[160]	2013	Implementation of coherent, evidence-based pathways in Danish rehabilitation practice	Denmark	Multiple settings		✓	✓	✓	Empirical study
Malte, McFall, et al.	[161]	2013	Survey of providers' attitudes toward integrating smoking cessation treatment into posttraumatic stress disorder care	USA	Hospital setting		✓	✓	✓	Empirical study
McCormack, McCarthy, et al.	[162]	2009	Development and testing of the Context Assessment Index (CAI)	Ireland, Northern Ireland, UK	Multiple settings		✓	✓	✓	Empirical study
McCullough, Chou, et al.	[163]	2015	The interplay of contextual elements in implementation: An ethnographic case study	USA	Multiple settings		✓	✓	✓	Empirical study
Palmcrantz, Tistad, et al.	[164]	2015	Assessing feasibility and acceptability of study procedures: getting ready for implementation of national stroke guidelines in out-patient health care	Sweden	Primary health care setting		✓	✓	✓	Empirical study
Schultz and Kitson	[165]	2010	Measuring the context of care in an Australian acute care hospital: A nurse survey	Australia	Hospital setting		✓	✓	✓	Empirical study
Stolee, Steeves, et al.	[166]	2010	Health information use in home care: Brainstorming barriers, facilitators, and recommendations	Canada	Home-based care		✓	✓	✓	Empirical study
Urquhart, Sargeant, et al.	[167]	2011	Factors related to the implementation and use of an innovation in cancer surgery	Canada	Hospital setting		✓	✓	✓	Empirical study
Watts, Shiner, et al.	[168]	2014	Implementation of evidence-based psychotherapies for posttraumatic stress disorder in VA specialty clinics	USA	Hospital setting		✓	✓	✓	Empirical study
Wente and Kleiber	[169]	2013	An Exploration of Context and the Use of Evidence-Based Nonpharmacological Practices in Emergency Departments	USA	Hospital setting		✓	✓	✓	Empirical study
Zubkoff, Carpenter-Song, et al.	[170]	2016	Clinicians’ Perception of Patient Readiness for Treatment: An Emerging Theme in Implementation Science?	USA	Primary health care setting		✓	✓	✓	Empirical study
Arling, Doebbeling, et al.	[171]	2011	Improving the implementation of evidence-based practice and information systems in healthcare: A social network approach	Canada	Multiple settings		✓	✓		Empirical study
Bahtsevani, Willman, et al.	[172]	2008	Developing an instrument for evaluating implementation of clinical practice guidelines: A test-retest study	Sweden	Hospital setting		✓	✓		Empirical study
Boaz, Baeza, et al.	[173]	2016	Does the "diffusion of innovations' model enrich understanding of research use? Case studies of the implementation of thrombolysis services for stroke	UK and Sweden	Hospital setting		✓	✓		Empirical study
Boström, Wallin, et al.	[174]	2007	Evidence-based practice and determinants of research use in elderly care in Sweden	Sweden	Community/Social care setting		✓	✓		Empirical study
Butow, Williams, et al.	[175]	2019	A psychological intervention (ConquerFear) for treating fear of cancer recurrence: Views of study therapists regarding sustainability	Australia	Hospital setting		✓	✓		Empirical study
Carlan, Kramer, et al.	[176]	2012	Digging into construction: Social networks and their potential impact on knowledge transfer	Canada	Construction setting		✓	✓		Empirical study
Chou, Graber, et al.	[177]	2018	Specifying an implementation framework for Veterans Affairs antimicrobial stewardship programmes: using a factor analysis approach	USA	Multiple settings		✓	✓		Empirical study
Conklin and Stolee	[178]	2008	A model for evaluating knowledge exchange in a network context	Canada	Community/Social care setting		✓	✓		Empirical study
Conklin, Lusk, et al.	[179]	2013	Knowledge brokers in a knowledge network: The case of Seniors Health Research Transfer Network knowledge brokers	Canada	Not reported		✓	✓		Empirical study
Cummings, Hutchinson, et al.	[180]	2010	The relationship between characteristics of context and research utilization in a pediatric setting	Canada	Hospital setting		✓	✓		Empirical study
Eldh, Fredriksson, et al.	[181]	2014	Facilitators and barriers to applying a national quality registry for quality improvement in stroke care	Sweden	Hospital setting		✓	✓		Empirical study
Elnitsky, Powell-Cope, et al.	[182]	2015	Implementation of Safe Patient Handling in the US Veterans Health System: A Qualitative Study of Internal Facilitators' Perceptions	USA	Hospital setting		✓	✓		Empirical study
Eriksson, Eriksson, et al.	[183]	2019	Occupational therapists’ perceptions of implementing a client-centered intervention in close collaboration with researchers: A mixed methods study	Sweden	multiple settings		✓	✓		Empirical study
Forberg, Unbeck, et al.	[184]	2016	Effects of computer reminders on complications of peripheral venous catheters and nurses' adherence to a guideline in paediatric care-a cluster randomised study	Sweden	Hospital setting		✓	✓		Empirical study
Gifford, Tavakoli, et al.	[185]	2015	Implementation of Smoking Cessation Treatment in VHA Substance Use Disorder Residential Treatment Programs	USA	Community/Social care setting		✓	✓		Empirical study
Hack, Ruether, et al.	[186]	2013	Promoting consultation recording practice in oncology: Identification of critical implementation factors and determination of patient benefit	Canada	Hospital setting		✓	✓		Empirical study
Hill, Guihan, et al.	[187]	2017	Use of the PARIHS Framework for Retrospective and Prospective Implementation Evaluations	USA	Multiple settings		✓	✓		Empirical study
Holt, Pankow, et al.	[188]	2018	Factors associated with using research evidence in national sport organisations	Canada	Not reported		✓	✓		Empirical study
Hurtubise, Rivard, et al.	[189]	2016	Virtual Knowledge Brokering: Describing the Roles and Strategies Used by Knowledge Brokers in a Pediatric Physiotherapy Virtual Community of Practice	Canada	Not reported		✓	✓		Empirical study
Hølge-Hazelton, Bruun, et al.	[190]	2019	Danish Translation and Adaptation of the Context Assessment Index With Implications for Evidence-Based Practice	Danmark	Hospital setting		✓	✓		Empirical study
Ismail, Squires, et al.	[191]	2018	The Influence of Context on Utilizing Research Evidence for Pain Management in Jordanian Pediatric Intensive Care Units (PICU)	Jordania	Hospital setting		✓	✓		Empirical study
Jansson and Forsberg	[192]	2016	How do nurses and ward managers perceive that evidence-based sources are obtained to inform relevant nursing interventions? - An exploratory study	Sweden	Hospital setting		✓	✓		Empirical study
Jansson, Pilhamar, et al.	[193]	2011	Factors and conditions that have an impact in relation to the successful implementation and maintenance of individual care plans	Sweden	Hospital setting		✓	✓		Empirical study
Kramer, Wells, et al.	[194]	2013	Did you have an impact? A theory-based method for planning and evaluating knowledge-transfer and exchange activities in occupational health and safety	Canada	Multiple settings		✓	✓		Empirical study
Lo, Hoben, et al.	[195]	2018	Importance of clinical educators to research use and suggestions for better efficiency and effectiveness: results of a cross-sectional survey of care aides in Canadian long-term care facilities	Canada	Community/Social care setting		✓	✓		Empirical study
Lundell, Tistad, et al.	[196]	2017	Building COPD care on shaky ground: A mixed methods study from Swedish primary care professional perspective	Sweden	Primary health care setting		✓	✓		Empirical study
McCalman, Tsey, et al.	[197]	2014	The characteristics, implementation and effects of Aboriginal and Torres Strait Islander health promotion tools: A systematic literature search	Not reported	Multiple settings		✓	✓		Empirical study
McKillop, Crisp, et al.	[198]	2012	Barriers and Enablers to Implementation of a New Zealand-Wide Guideline for Assessment and Management of Cardiovascular Risk in Primary Health Care: A Template Analysis	New Zealand	Primary health care setting		✓	✓		Empirical study
Meherali, Paul, et al.	[199]	2017	Use of Research by Undergraduate Nursing Students: A Qualitative Descriptive Study	Canada	Not reported		✓	✓		Empirical study
Naik, Lawrence, et al.	[200]	2015	Building a primary care/research partnership: Lessons learned from a telehealth intervention for diabetes and depression	USA	Primary health care setting		✓	✓		Empirical study
Nygårdh, Ahlström, et al.	[201]	2016	Handling a challenging context: Experiences of facilitating evidence-based elderly care	Sweden	Community/Social care setting		✓	✓		Empirical study
Peirson, Ciliska, et al.	[202]	2012	Building capacity for evidence informed decision making in public health: A case study of organizational change	Canada	Primary health care setting		✓	✓		Empirical study
Perry, Bellchambers, et al.	[203]	2011	Examination of the utility of the Promoting Action on Research Implementation in Health Services framework for implementation of evidence based practice in residential aged care settings	Australia	Community/Social care setting		✓	✓		Empirical study
Sandström, Willman, et al.	[204]	2015	Perceptions of national guidelines and their (non) implementation in mental healthcare: A deductive and inductive content analysis	Sweden	Multiple settings		✓	✓		Empirical study
Sharp, Pineros, et al.	[205]	2004	A qualitative study to identify barriers and facilitators to implementation of pilot interventions in the Veterans Health Administration (VHA) Northwest network	USA	Hospital setting		✓	✓		Empirical study
Stetler, Legro, et al.	[206]	2006	Role of "external facilitation" in implementation of research findings: A qualitative evaluation of facilitation experiences in the Veterans Health Administration	USA	Not reported		✓	✓		Empirical study
Stevens, Riahi, et al.	[207]	2011	The Influence of Context on Pain Practices in the NICU: Perceptions of Health Care Professionals	Canada	Hospital setting		✓	✓		Empirical study
Ullrich, Lavela, et al.	[208]	2014	Associations between perceptions of evidence and adoption of H1N1 influenza infection prevention strategies among healthcare workers providing care to persons with spinal cord injury	USA	Multiple settings		✓	✓		Empirical study
Ullrich, Sahay, et al.	[209]	2014	Use of implementation theory: A focus on PARIHS	USA	Multiple settings		✓	✓		Empirical study
Vabo, Slettebø, et al.	[210]	2017	Participants' perceptions of an intervention implemented in an Action Research Nursing Documentation Project	Norway	Multiple settings		✓	✓		Empirical study
Wallin, Estabrooks, et al.	[211]	2006	Development and validation of a derived measure of research utilization by nurses	Canada	Not reported		✓	✓		Empirical study
Ward, Baloh, et al.	[212]	2017	Promoting Action on Research Implementation in Health Services framework applied to TeamSTEPPS implementation in small rural hospitals	USA	Hospital setting		✓	✓		Empirical study
Wilde, Sonley, et al.	[213]	2019	Mindfulness Training in UK Secondary Schools: a Multiple Case Study Approach to Identification of Cornerstones of Implementation	UK	Not reported		✓	✓		Empirical study
Øye, Mekki, et al.	[214]	2015	Evidence Molded by Contact with Staff Culture and Patient Milieu: an Analysis of the Social Process of Knowledge Utilization in Nursing Homes	Norway	Community/Social care setting		✓	✓		Empirical study
Benoit and Semenic	[215]	2014	Barriers and Facilitators to Implementing the Baby-Friendly Hospital Initiative in Neonatal Intensive Care Units	Canada	Hospital setting		✓		✓	Empirical study
Campbell and Profetto-McGrath	[216]	2013	Skills and Attributes Required by Clinical Nurse Specialists to Promote Evidence-Based Practice	Canada	Multiple settings		✓		✓	Empirical study
Douglas, Hinckley, et al.	[217]	2014	Perceptions of speech-language pathologists linked to evidence-based practice use in skilled nursing facilities	USA	Community/Social care setting		✓		✓	Empirical study
Gunningberg, Brudin, et al.	[218]	2010	Nurse Managers' prerequisite for nursing development: A survey on pressure ulcers and contextual factors in hospital organizations	Sweden	Multiple settings		✓		✓	Empirical study
Hagedorn and Heideman	[219]	2010	The relationship between baseline Organizational Readiness to Change Assessment subscale scores and implementation of hepatitis prevention services in substance use disorders treatment clinics: A case study	USA	Multiple settings		✓		✓	Empirical study
Henry Dr, Hagedorn, et al.	[220]	2010	A formative evaluation of organizational readiness to implement nurse-initiated HIV rapid testing in two veterans health administration substance use disorder clinics	USA	Not reported		✓		✓	Empirical study
Hälleberg Nyman, Forsman, et al.	[221]	2019	Promoting evidence-based urinary incontinence management in acute nursing and rehabilitation care—A process evaluation of an implementation intervention in the orthopaedic context	Sweden	Hospital setting		✓		✓	Empirical study
Jacobsen, Mekki, et al.	[222]	2017	A mixed method study of an education intervention to reduce use of restraint and implement person-centered dementia care in nursing homes	Norway	Community/Social care setting		✓		✓	Empirical study
Kirkpatrick, Boblin, et al.	[223]	2014	The nurse as bricoleur in falls prevention: Learning from a case study of the implementation of fall prevention best practices	Canada	Hospital setting		✓		✓	Empirical study
Stryczek, Lea, et al.	[224]	2019	De-implementing Inhaled Corticosteroids to Improve Care and Safety in COPD Treatment: Primary Care Providers’ Perspectives	USA	Primary health care setting		✓		✓	Empirical study
Bokhour, Saifu, et al.	[225]	2015	The role of evidence and context for implementing a multimodal intervention to increase HIV testing	USA	Primary health care setting		✓			Empirical study
Garvin, Kim, et al.	[226]	2018	Automating Quality Measures for Heart Failure Using Natural Language Processing: A Descriptive Study in the Department of Veterans Affairs	USA	Multiple settings		✓			Empirical study
Harrison, Reddy, et al.	[227]	2019	Implementing an Inpatient Acupuncture Service for Pain and Symptom Management: Identifying Opportunities and Challenges	USA	Hospital setting		✓			Empirical study
Hawkins, Malte, et al.	[228]	2017	Survey of Primary Care and Mental Health Prescribers' Perspectives on Reducing Opioid and Benzodiazepine Co-Prescribing Among Veterans	USA	Multiple settings		✓			Empirical study
Hoben, Estabrooks, et al.	[229]	2016	Factor Structure, Reliability and Measurement Invariance of the Alberta Context Tool and the Conceptual Research Utilization Scale, for German Residential Long Term Care	Germany	Community/Social care setting		✓			Empirical study
Hofler, Cordes, et al.	[230]	2017	Implementing Immediate Postpartum Long-Acting Reversible Contraception Programs	USA	Hospital setting		✓			Empirical study
Hommel, Gunningberg, et al.	[231]	2017	Successful factors to prevent pressure ulcers – an interview study	Sweden	Hospital setting		✓			Empirical study
Kothari, Boyko, et al.	[232]	2015	Communities of practice for supporting health systems change: A missed opportunity	Canada	Community/Social care setting		✓			Empirical study
Kristensen, Borg, et al.	[233]	2012	Aspects affecting occupational therapists' reasoning when implementing research-based evidence in stroke rehabilitation	Denmark	Multiple settings		✓			Empirical study
McCullough, Gillespie, et al.	[234]	2017	Forming and activating an internal facilitation group for successful implementation: A qualitative study	USA	Not reported		✓			Empirical study
Mocumbi, McKee, et al.	[235]	2018	Ready to deliver maternal and newborn care? Health providers’ perceptions of their work context in rural Mozambique	Mozambique	Primary health care setting		✓			Empirical study
Murphy, Washington, et al.	[236]	2019	Identifying and Addressing Language Needs in Primary Care: a Pilot Implementation Study	USA	Primary health care setting		✓			Empirical study
Rycroft-Malone, Fontenla, et al.	[237]	2010	A realistic evaluation: The case of protocol-based care	UK	Not reported		✓			Empirical study
Shimada, Hogan, et al.	[238]	2013	Patient-provider secure messaging in VA: Variations in adoption and association with urgent care utilization	USA	Multiple settings		✓			Empirical study
Stolee, Hiller, et al.	[239]	2012	Flying by the Seat of Our Pants: Current Processes to Share Best Practices to Deal With Elder Abuse	Canada	Not reported		✓			Empirical study
Tukey, Clark, et al.	[240]	2016	Readiness for Implementation of Lung Cancer Screening A National Survey of Veterans Affairs Pulmonologists	USA	Hospital setting		✓			Empirical study
Xiang, Robinson-Lane, et al.	[241]	2018	Implementing and sustaining evidence-based practice in health care: The Bridge Model experience	USA	Community/Social care setting		✓			Empirical study
Zubkoff, Shiner, et al.	[242]	2016	Staff Perceptions of Substance Use Disorder Treatment in VA Primary Care–Mental Health Integrated Clinics	USA	Primary health care setting		✓			Empirical study
Estabrooks, Kenny, et al.	[243]	2007	A comparison of research utilization among nurses working in Canadian civilian and United States Army healthcare settings	Canada, USA	Hospital setting			✓	✓	Empirical study
Ireland, Kirkpatrick, et al.	[244]	2013	The real world journey of implementing fall prevention best practices in three acute care hospitals: A case study	Canada	Hospital setting			✓	✓	Empirical study
Kristensen, Borg, et al.	[245]	2011	Facilitation of research-based evidence within occupational therapy in stroke rehabilitation	Denmark	Multiple settings			✓	✓	Empirical study
Lavoie-Tremblay, Richer, et al.	[246]	2012	Implementation of Evidence-Based Practices in the Context of a Redevelopment Project in a Canadian Healthcare Organization	Canada	Hospital setting			✓	✓	Empirical study
Leclair, Ripat, et al.	[247]	2013	Advancing the use of theory in occupational therapy: A collaborative process	Canada	Not reported			✓	✓	Empirical study
Matthew-Maich, Ploeg, et al.	[248]	2013	Supporting the uptake of nursing guidelines: What you really need to know to move nursing guidelines into practice	Canada	Hospital setting			✓	✓	Empirical study
Ranse, Yates, et al.	[249]	2015	Factors influencing the provision of end-of-life care in critical care settings: Development and testing of a survey instrument	Australia	Hospital setting			✓	✓	Empirical study
Squires, Estabrooks, et al.	[250]	2013	The influence of organizational context on the use of research by nurses in Canadian pediatric hospitals	Canada	Hospital setting			✓	✓	Empirical study
Svensson, Ohlsson, et al.	[251]	2012	Development and implementation of a standardized care plan for carotid endarterectomy	Sweden	Hospital setting			✓	✓	Empirical study
Tierney, Kislov, et al.	[252]	2014	A qualitative study of a primary-care based intervention to improve the management of patients with heart failure: The dynamic relationship between facilitation and context	UK	Primary health care setting			✓	✓	Empirical study
Urquhart, Porter, et al.	[253]	2014	Multi-level factors influence the implementation and use of complex innovations in cancer care: A multiple case study of synoptic reporting	Canada	Multiple settings			✓	✓	Empirical study
Warner	[254]	2013	Synthesizing research evidence for therapists providing home-based rehabilitative care	Canada	Home-based care			✓	✓	Empirical study
Warner and Stadnyk	[255]	2014	What is the evidence and context for implementing family-centered care for older adults	Canada	Home-based care			✓	✓	Empirical study
Wilson, Sleutel, et al.	[256]	2015	Empowering nurses with evidence-based practice environments: Surveying magnet®, pathway to excellence®, and non-magnet facilities in one healthcare system	USA	Hospital setting			✓	✓	Empirical study
Abrahamson, Miech, et al.	[257]	2015	Pay-for-performance policy and data-driven decision making within nursing homes: A qualitative study	USA	Community/Social care setting			✓		Empirical study
Backman, Hebert, et al.	[258]	2018	Implementation of a multimodal patient safety improvement program "SafetyLEAP" in intensive care units: A cross-case study analysis	Canada	Hospital setting			✓		Empirical study
Brobeck, Odencrants, et al.	[259]	2013	Health promotion practice and its implementation in Swedish health care	Sweden	Primary health care setting			✓		Empirical study
Brown and McCormack	[260]	2011	Developing the practice context to enable more effective pain management with older people: An action research approach	UK	Hospital setting			✓		Empirical study
Carlfjord, Andersson, et al.	[261]	2012	Applying the RE-AIM framework to evaluate two implementation strategies used to introduce a tool for lifestyle intervention in Swedish primary health care	Sweden	Primary health care setting			✓		Empirical study
Carlfjord, Kristenson, et al.	[262]	2011	Experiences of Working with the Tobacco Issue in the Context of Health Promoting Hospitals and Health Services: A Qualitative Study	Sweden	Multiple settings			✓		Empirical study
Cranley, Birdsell, et al.	[263]	2012	Insights into the impact and use of research results in a residential long-term care facility: a case study	Canada	Community/Social care setting			✓		Empirical study
Dogherty, Harrison, et al.	[264]	2013	Turning Knowledge Into Action at the Point-of-Care: The Collective Experience of Nurses Facilitating the Implementation of Evidence-Based Practice	Canada	Multiple settings			✓		Empirical study
Espirito Santo and Choquette	[265]	2013	Experience of adapting and implementing an evidence-based nursing guideline for prevention of diaper dermatitis in a paediatric oncology setting	Canada	Hospital setting			✓		Empirical study
Harvey, Kitson, et al.	[266]	2012	Promoting continence in nursing homes in four European countries: The use of PACES as a mechanism for improving the uptake of evidence-based recommendations	Republic Ireland, Sweden, UK, Netherlands	Community/Social care setting			✓		Empirical study
Hermansyah, Sainsbury, et al.	[267]	2017	The operation of a Research and Development (R&D) program and its significance for practice change in community pharmacy	Australia	Not reported			✓		Empirical study
Johnston, Gagnon, et al.	[268]	2007	One-on-One Coaching to Improve Pain Assessment and Management Practices of Pediatric Nurses	Canada	Hospital setting			✓		Empirical study
Kinley, Denton, et al.	[269]	2018	Development and implementation of the Steps to Successful Palliative Care programme in residential care homes for people with a learning disability	UK	Community/Social care setting			✓		Empirical study
Kinley, Denton, et al.	[270]	2018	Improving the approach to future care planning in care homes	UK	Community/Social care setting			✓		Empirical study
Marfurt-Russenberger, Axelin, et al.	[271]	2016	The Experiences of Professionals Regarding Involvement of Parents in Neonatal Pain Management	Switzerland	Hospital setting			✓		Empirical study
Meagher-Stewart, Solberg, et al.	[272]	2012	Understanding the role of communities of practice in evidence-informed decision making in public health	Canada	Primary health care setting			✓		Empirical study
Meyer-Zehnder, Albisser Schleger, et al.	[273]	2017	How to introduce medical ethics at the bedside - Factors influencing the implementation of an ethical decision-making model	Switzerland	Hospital setting			✓		Empirical study
Nguyen and Wilson	[274]	2016	Hospital readiness for undertaking evidence-based practice: A survey	Vietnam	Hospital setting			✓		Empirical study
Painter, Clark, et al.	[275]	2014	Physical function and physical activity assessment and promotion in the hemodialysis clinic: A qualitative study	USA	Hospital setting			✓		Empirical study
Powell-Cope, Moore, et al.	[276]	2015	Perceptions of Practice Guidelines for People with Spinal Cord Injury	USA	Multiple settings			✓		Empirical study
Powrie, Danly, et al.	[277]	2014	Using implementation science to facilitate evidence-based practice changes to promote optimal outcomes for orthopaedic patients	USA	Hospital setting			✓		Empirical study
Scott, Estabrooks, et al.	[278]	2008	A context of uncertainty: How context shapes nurses' research utilization behaviors	Canada	Hospital setting			✓		Empirical study
Skene, Gerrish, et al.	[279]	2019	Developing family-centred care in a neonatal intensive care unit: An action research study	UK	Hospital setting			✓		Empirical study
Squires, Aloisio, et al.	[280]	2019	Attributes of context relevant to healthcare professionals' use of research evidence in clinical practice: a multi-study analysis	Canada, Australia	Multiple settings			✓		Empirical study
Stenberg and Wann-Hansson	[281]	2011	Health care professionals' attitudes and compliance to clinical practice guidelines to prevent falls and fall injuries	Sweden	Hospital setting			✓		Empirical study
Thunberg, Ferm, et al.	[282]	2019	Implementation of pictorial support for communication with people who have been forced to flee: Experiences from neonatal care	Sweden	Hospital setting			✓		Empirical study
Tishelman, Bergenmar, et al.	[283]	2008	Using undergraduate nursing students as mediators in a knowledge transfer programme for care for patients with advanced cancer	Sweden	Multiple settings			✓		Empirical study
Wedge and Gosney	[284]	2005	Pressure-relieving equipment: Promoting its correct use amongst nurses via differing modes of educational delivery	UK	Hospital setting			✓		Empirical study
Anderson, Wang, et al.	[285]	2012	Comprehensive assessment of chronic pain management in primary care: A first phase of a quality improvement initiative at a multisite community health center	USA	Primary health care setting				✓	Empirical study
Arce, De Ormijana, et al.	[286]	2014	A qualitative study on clinicians' perceptions about the implementation of a population risk stratification tool in primary care practice of the Basque Health Service	Spain	Primary health care setting				✓	Empirical study
Bergström, Skeen, et al.	[287]	2015	Health system context and implementation of evidence-based practices-development and validation of the Context Assessment for Community Health (COACH) tool for low- and middle-income settings	South Africa, Vietnam, Uganda, Nicaragua, Bangladesh	Multiple settings				✓	Empirical study
Bramley, Manning, et al.	[288]	2018	Engaging and developing front-line clinical nurses to drive care excellence: Evaluating the Chief Nurse Excellence in Care Junior Fellowship initiative	UK	Hospital setting				✓	Empirical study
Capasso, Collins, et al.	[289]	2009	Outcomes of a clinical nurse specialist-initiated wound care education program: Using the promoting action on research implementation in health services framework	USA	Hospital setting				✓	Empirical study
Conklin, Cohen-Schneider, et al.	[290]	2012	Enacting change through action learning: Mobilizing and managing power and emotion	Canada	Hospital setting				✓	Empirical study
Curran, Woo, et al.	[291]	2015	Training Substance Use Disorder Counselors in Cognitive Behavioral Therapy for Depression: Development and Initial Exploration of an Online Training Program	USA	Primary health care setting				✓	Empirical study
Damschroder, Moin, et al.	[292]	2015	Implementation and evaluation of the VA DPP clinical demonstration: protocol for a multi-site non-randomized hybrid effectiveness-implementation type III trial	USA	Multiple settings				✓	Empirical study
Doran, Haynes, et al.	[293]	2010	Supporting Evidence-Based Practice for Nurses through Information Technologies	Canada	Multiple settings				✓	Empirical study
Estrada	[294]	2009	Exploring Perceptions of a Learning Organization by RNs and Relationship to EBP Beliefs and Implementation in the Acute Care Setting	USA	Hospital setting				✓	Empirical study
Fitzgerald and Harvey	[295]	2015	Translational networks in healthcare? Evidence on the design and initiation of organizational networks for knowledge mobilization	UK	Multiple settings				✓	Empirical study
Forgeron, Jongudomkarn, et al.	[296]	2009	Children's pain assessment in Northeastern Thailand: Perspectives of health professionals	Thailand	Hospital setting				✓	Empirical study
Friberger and Falkman	[297]	2013	Collaboration processes, outcomes, challenges and enablers of distributed clinical communities of practice	Sweden	Not reported				✓	Empirical study
Graham, Maddox, et al.	[298]	2013	Coronary Stents and Subsequent Surgery: Reported Provider Attitudes and Practice Patterns	USA	Hospital setting				✓	Empirical study
Hefter and Gerson	[299]	2010	Increasing Adherence to Scheduled Outpatient Dobutamine Stress Echocardiograms	USA	Hospital setting				✓	Empirical study
Hunter, Chinman, et al.	[300]	2009	Technical assistance as a prevention capacity-building tool: A demonstration using the getting to outcomes® framework	USA	Community/Social care setting				✓	Empirical study
Mallidou, Cummings, et al.	[301]	2011	Staff, space, and time as dimensions of organizational slack: A psychometric assessment	Canada	Hospital setting				✓	Empirical study
Nilsson Kajermo, Böe, et al.	[302]	2013	Swedish Translation, Adaptation and Psychometric Evaluation of the Context Assessment Index (CAI)	Sweden	Multiple settings				✓	Empirical study
Powers, Preshong, et al.	[303]	2016	A Model of Regulatory Alignment to Enhance the Long-Term Care Survey Process in a Veterans Health Care Network	USA	Not reported				✓	Empirical study
Ryan, Franklin, et al.	[304]	2018	Ranking and prioritizing strategies for reducing mortality and morbidity from noncommunicable diseases post disaster: An Australian perspective	Australia	Community/Social care setting				✓	Empirical study
Shuman, Ploutz-Snyder, et al.	[305]	2018	Development and Testing of the Nurse Manager EBP Competency Scale	USA	Hospital setting				✓	Empirical study
Shuman, Powers, et al.	[306]	2019	Unit Leadership and Climates for Evidence-Based Practice Implementation in Acute Care: A Cross-Sectional Descriptive Study	USA	Hospital setting				✓	Empirical study
Siraj-Blatchford, Taggart, et al.	[307]	2008	Towards the transformation of practice in early childhood education: The effective provision of pre-school education (EPPE) project	UK	Multiple settings				✓	Empirical study
Squires, Hayduk, et al.	[308]	2015	Reliability and validity of the Alberta context tool (ACT) with professional nurses: Findings from a multi-study analysis	Canada, Australia	Multiple settings				✓	Empirical study
Tucker, Olson, et al.	[309]	2009	Evidence-Based Practice Self-efficacy Scale: Preliminary Reliability and Validity	USA	Multiple settings				✓	Empirical study
Urquhart, Jackson, et al.	[310]	2015	Health system-level factors influence the implementation of complex innovations in cancer care	Canada	Multiple settings				✓	Empirical study
Vetter	[311]	2015	The Influence of Clinical Decision Support on Diagnostic Accuracy in Nurse Practitioners	USA	Home-based care				✓	Empirical study
Weaver, Smith, et al.	[312]	2007	Interventions to increase influenza vaccination rates in veterans with spinal cord injuries and disorders	USA	Hospital setting				✓	Empirical study
Liedgren, Elvhage, et al.	[313]	2016	The Use of Decision Support Systems in Social Work: A Scoping Study Literature Review	Sweden	Multiple settings		✓	✓	✓	Empirical review study
Brown and McCormack	[314]	2005	Developing postoperative pain management: Utilising the promoting action on research implementation in health services (PARIHS) framework	Not reported	Hospital setting		✓	✓		Empirical review study
Helfrich, Damschroder, et al.	[13]	2010	A critical synthesis of literature on the promoting action on research implementation in health services (PARIHS) framework	Not reported	Not reported		✓	✓		Empirical review study
McCalman, Bainbridge, et al.	[315]	2016	The effectiveness of implementation in Indigenous Australian healthcare: An overview of literature reviews	Not reported	Multiple settings		✓	✓		Empirical review study
Meijers, Janssen, et al.	[316]	2006	Assessing the relationships between contextual factors and research utilization in nursing: systematic literature review	Not reported	Not reported		✓	✓		Empirical review study
Milner, Estabrooks, et al.	[317]	2006	Research utilization and clinical nurse educators: A systematic review	Not reported	Not reported		✓	✓		Empirical review study
Pfadenhauer, Gerhardus, et al.	[318]	2017	Making sense of complexity in context and implementation: The Context and Implementation of Complex Interventions (CICI) framework	Not reported	Not reported		✓	✓		Empirical review study
Toms, Williams, et al.	[319]	2019	The development and theoretical application of an implementation framework for dialectical behaviour therapy: a critical literature review	Sweden, USA, UK, Australia, Ireland, New Zealand, Canada, Netherlands, Germany	Not reported		✓	✓		Empirical review study
Wood, Migliore, et al.	[320]	2019	Confronting Challenges in Reducing Heart Failure 30-Day Readmissions: Lessons Learned With Implications for Evidence-Based Practice	USA	Hospital setting		✓	✓		Empirical review study
Franx, Dixon, et al.	[321]	2013	Implementation strategies for collaborative primary care-mental health models	Netherlands, USA, and UK	Multiple settings		✓			Empirical review study
Hudon, Gervais, et al.	[322]	2015	The contribution of conceptual frameworks to knowledge translation interventions in physical therapy	Canada	Not reported		✓			Empirical review study
Dogherty, Harrison, et al.	[323]	2010	Facilitation as a role and process in achieving evidence-based practice in nursing: A focused review of concept and meaning	Not reported	Multiple settings			✓	✓	Empirical review study
Flottorp, Oxman, et al.	[324]	2013	A checklist for identifying determinants of practice: A systematic review and synthesis of frameworks and taxonomies of factors that prevent or enable improvements in healthcare professional practice	Not reported	Not reported			✓	✓	Empirical review study
Aas, Tuntland, et al.	[325]	2011	Workplace interventions for neck pain in workers	Netherlands, Norway, Finland, Sweden, USA	Not reported			✓		Empirical review study
Geerligs, Rankin, et al.	[326]	2018	Hospital-based interventions: A systematic review of staff-reported barriers and facilitators to implementation processes	USA, UK, Canada, Australia/ New Zealand, Denmark, Sweden, Finland, Italy, the Netherlands, Uganda, South Africa, Tanzania, Ghana, Mexico	Multiple settings			✓		Empirical review study
McConnell, O'Halloran, et al.	[327]	2013	Systematic Realist Review of Key Factors Affecting the Successful Implementation and Sustainability of the Liverpool Care Pathway for the Dying Patient	UK	Multiple settings			✓		Empirical review study
Rogers	[328]	2009	Transferring research into practice: An integrative review	Not reported	Not reported			✓		Empirical review study
Salter and Kothari	[329]	2014	Using realist evaluation to open the black box of knowledge translation: A state-of-the-art review	UK	Multiple settings			✓		Empirical review study
Sandström, Borglin, et al.	[330]	2011	Promoting the Implementation of Evidence-Based Practice: A Literature Review Focusing on the Role of Nursing Leadership	Not reported	Multiple settings			✓		Empirical review study
Wahr, Abernathy, et al.	[331]	2017	Medication safety in the operating room: literature and expert-based recommendations	USA	Not reported			✓		Empirical review study
Baskerville, Liddy, et al.	[332]	2012	Systematic Review and Meta-Analysis of Practice Facilitation Within Primary Care Settings	23 studies from various countries, not described in the paper.	Primary health care setting				✓	Empirical review study
Colquhoun, Letts, et al.	[333]	2010	A scoping review of the use of theory in studies of knowledge translation	Several countries were represented in this review study but not clearly stated.	Multiple settings				✓	Empirical review study
Leeman, Calancie, et al.	[334]	2017	Developing Theory to Guide Building Practitioners' Capacity to Implement Evidence-Based Interventions	USA, UK, Canada	Not reported				✓	Empirical review study
Nilsen and Bernhardsson	[335]	2019	Context matters in implementation science: A scoping review of determinant frameworks that describe contextual determinants for implementation outcomes	N/A	Not reported				✓	Empirical review study
O'Keefe-McCarthy, Santiago, et al.	[336]	2008	Ventilator-associated pneumonia bundled strategies: An evidence-based practice	Canada	Hospital setting				✓	Empirical review study
Prihodova, Guerin, et al.	[337]	2019	Key components of knowledge transfer and exchange in health services research: Findings from a systematic scoping review	N/A	Multiple settings				✓	Empirical review study
Tabak, Khoong, et al.	[3]	2012	Bridging research and practice: Models for dissemination and implementation research	Not reported	Not reported				✓	Empirical review study
Ward, House, et al.	[338]	2009	Developing a framework for transferring knowledge into action: a thematic analysis of the literature	Not reported	Multiple settings				✓	Empirical review study
Doran and Sidani	[339]	2007	Outcomes-focused knowledge translation: A framework for knowledge translation and patient outcomes improvement	N/A	Not reported	✓	✓	✓		Opinion/ theoretical paper
Ritchie, Dollar, et al.	[340]	2014	Responding to needs of clinical operations partners: Transferring implementation facilitation knowledge and skills	N/A	Primary health care setting	✓				Opinion/ theoretical paper
Damschroder, Aron, et al.	[341]	2009	Fostering implementation of health services research findings into practice: A consolidated framework for advancing implementation science	N/A	Not reported		✓	✓		Opinion/ theoretical paper
Florczak	[342]	2016	Evidence-Based Practice: What’s New Is Old	N/A	Not reported		✓	✓		Opinion/ theoretical paper
Kavanagh, Stevens, et al.	[343]	2008	Examining appreciative inquiry as a knowledge translation intervention in pain management	N/A	Not reported		✓	✓		Opinion/ theoretical paper
Kavanagh, Watt-Watson, et al.	[344]	2007	An examination of the factors enabling the successful implementation of evidence-based acute pain practices into pediatric nursing	N/A	Multiple settings		✓	✓		Opinion/ theoretical paper
Rongey, Asch, et al.	[345]	2011	Access to care for vulnerable veterans with hepatitis C: A hybrid conceptual framework and a case study to guide translation	N/A	Multiple settings		✓	✓		Opinion/ theoretical paper
Rycroft-Malone	[346]	2007	Theory and knowledge translation: Setting some coordinates	N/A	Not reported		✓	✓		Opinion/ theoretical paper
Stetler, Damschroder, et al.	[347]	2011	A Guide for applying a revised version of the PARIHS framework for implementation	N/A	Not reported		✓	✓		Opinion/ theoretical paper
Tucker, Klotzbach, et al.	[348]	2006	Lessons learned in translating research evidence on early intervention programs into clinical care	N/A	Not reported		✓	✓		Opinion/ theoretical paper
Urquhart, Sargeant, et al.	[349]	2013	Exploring the usefulness of two conceptual frameworks for understanding how organizational factors influence innovation implementation in cancer care	N/A	Not reported		✓	✓		Opinion/ theoretical paper
Wallin, Profetto-McGrath, et al.	[350]	2005	Implementing nursing practice guidelines. A complex undertaking	N/A	Not reported		✓	✓		Opinion/ theoretical paper
Owen and Milburn	[351]	2001	Implementing research findings into practice: Improving and developing services for women with serious and enduring mental health problems	N/A	Community/Social care setting		✓		✓	Opinion/ theoretical paper
Blackwood	[352]	2003	Can protocolised-weaning developed in the United States transfer to the United Kingdom context: A discussion	N/A	Hospital setting		✓			Opinion/ theoretical paper
Gawlinski and Rutledge	[353]	2008	Selecting a model for evidence-based practice changes: A practical approach	N/A	Hospital setting		✓			Opinion/ theoretical paper
Genuis	[354]	2007	Evolving information in an evidence-Based world: Theoretical considerations	N/A	Not reported		✓			Opinion/ theoretical paper
Hunt, Curran, et al.	[355]	2012	Partnership for implementation of evidence-based mental health practices in rural federally qualified health centers: Theory and methods	N/A	Community/Social care setting		✓			Opinion/ theoretical paper
Nilsen	[2]	2015	Making sense of implementation theories, models and frameworks	N/A	Not reported		✓			Opinion/ theoretical paper
Pfadenhauer, Mozygemba, et al.	[356]	2015	Context and implementation: A concept analysis towards conceptual maturity	N/A	Not reported		✓			Opinion/ theoretical paper
Ruth and Matusitz	[357]	2013	Comparative Standards of Evidence in Social Work	N/A	Community/Social care setting		✓			Opinion/ theoretical paper
Squires, Reay, et al.	[358]	2012	Designing strategies to implement research-based policies and procedures: A set of recommendations for nurse leaders based on the PARiHS framework	N/A	Not reported		✓			Opinion/ theoretical paper
Harvey and Kitson	[359]	2016	PARIHS revisited: From heuristic to integrated framework for the successful implementation of knowledge into practice	N/A	Not reported			✓	✓	Opinion/ theoretical paper
Larkin, Griffith, et al.	[360]	2007	Promoting research utilization using a conceptual framework	N/A	Hospital setting			✓	✓	Opinion/ theoretical paper
Spassiani, Parker Harris, et al.	[361]	2016	Exploring How Knowledge Translation Can Improve Sustainability of Community-based Health Initiatives for People with Intellectual/Developmental Disabilities	N/A	Not reported			✓	✓	Opinion/ theoretical paper
Andrews and Moon	[362]	2005	Space, place, and the evidence base: Part II - Rereading nursing environment through geographical research	N/A	Hospital setting			✓		Opinion/ theoretical paper
Andrews, Holmes, et al.	[363]	2005	'Airplanes are flying nursing homes': geographies in the concepts and locales of gerontological nursing practice	N/A	Community/Social care setting			✓		Opinion/ theoretical paper
Bucknall	[364]	2007	A gaze through the lens of decision theory toward knowledge translation science	N/A	Not reported			✓		Opinion/ theoretical paper
Gibson	[365]	2005	Evidence in action: Fostering growth of research-based practice in children's cancer nursing	N/A	Multiple settings			✓		Opinion/ theoretical paper
Bandeira, Witt, et al.	[366]	2017	The use of a methodological framework in the implementation of evidence as part of nursing research	N/A	Not reported				✓	Opinion/ theoretical paper
Boucher, Roper, et al.	[367]	2013	Science and Practice Aligned Within Nursing Structure and Process for Evidence-Based Practice	N/A	Multiple settings				✓	Opinion/ theoretical paper
Chambers, Luesby, et al.	[368]	2010	The Seniors Health Research Transfer Network knowledge network model: System-wide implementation for health and healthcare of seniors	N/A	Multiple settings				✓	Opinion/ theoretical paper
Doane, Reimer-Kirkham, et al.	[369]	2015	(Re)theorizing integrated knowledge translation a heuristic for knowledge-as-action	N/A	Not reported				✓	Opinion/ theoretical paper
Ellen, Panisset, et al.	[370]	2017	A Knowledge Translation framework on ageing and health	N/A	Not reported				✓	Opinion/ theoretical paper
Harvey, Fitzgerald, et al.	[371]	2011	The NIHR collaboration for leadership in applied health research and care (CLAHRC) for Greater Manchester: Combining empirical, theoretical and experiential evidence to design and evaluate a large-scale implementation strategy	N/A	Multiple settings				✓	Opinion/ theoretical paper
Hutchinson, Wilkinson, et al.	[372]	2012	Using the Promoting Action on Research Implementation in Health Services Framework to Guide Research Use in the Practice Setting	N/A	Not reported				✓	Opinion/ theoretical paper
Hysong, Woodard, et al.	[373]	2014	Publishing Protocols for Partnered Research	N/A	Multiple settings				✓	Opinion/ theoretical paper
Jeffs, Sidani, et al.	[374]	2013	Using theory and evidence to drive measurement of patient, nurse and organizational outcomes of professional nursing practice	N/A	Not reported				✓	Opinion/ theoretical paper
Jukes and Aspinall	[375]	2015	Leadership and learning disability nursing	N/A	Not reported				✓	Opinion/ theoretical paper
Lynch, Mudge, et al.	[376]	2018	There is nothing so practical as a good theory: a pragmatic guide for selecting theoretical approaches for implementation projects	N/A	Multiple settings				✓	Opinion/ theoretical paper
Matthew-Maich, Ploeg, et al.	[377]	2010	Transformative learning and research utilization in nursing practice: A missing link?	N/A	Not reported				✓	Opinion/ theoretical paper
Mitchell, Fisher, et al.	[378]	2010	A thematic analysis of theoretical models for translational science in nursing: Mapping the field	N/A	Not reported				✓	Opinion/ theoretical paper
O’Meara, Furness, et al.	[379]	2017	Educating paramedics for the future: A holistic approach	N/A	Not reported				✓	Opinion/ theoretical paper
Persaud	[380]	2014	Enhancing learning, innovation, adaptation, and sustainability in health care organizations: The ELIAS performance management framework	N/A	Not reported				✓	Opinion/ theoretical paper
Schoville and Titler	[381]	2015	Guiding Healthcare Technology Implementation: A New Integrated Technology Implementation Model	N/A	Not reported				✓	Opinion/ theoretical paper
Shah, Warre, et al.	[382]	2013	Quality improvement initiatives in neonatal intensive care unit networks: Achievements and challenges	N/A	Hospital setting				✓	Opinion/ theoretical paper
Smith	[383]	2018	Revisiting implementation theory: An interdisciplinary comparison between urban planning and healthcare implementation research	N/A	Not reported				✓	Opinion/ theoretical paper
Tilson and Mickan	[144]	2014	Promoting physical therapists' of research evidence to inform clinical practice: Part 1 - Theoretical foundation, evidence, and description of the PEAK program	N/A	Multiple settings				✓	Opinion/ theoretical paper
Warner and Townsend	[384]	2012	Applying knowledge translation theories to occupation	N/A	Not reported				✓	Opinion/ theoretical paper
Young	[385]	2015	Solving the wicked problem of hospital malnutrition	N/A	Not reported				✓	Opinion/ theoretical paper

^a^Protocols planned to use PARIHS in the analysis

Of these 367 articles, 235 cited Kitson et al. [5], 208 cited Kitson et al. [18], 136 cited Rycroft-Malone et al. [8], and 92 cited Rycroft-Malone et al. [9]. In total, the 367 articles consisted of 35 protocols [25, 28, 29, 31–38, 40, 42, 44–50, 52–54, 56, 57]. A further 255 articles reported empirical studies:

▪ 91 where PARIHS guided the development of the intervention [58–82, 84–143, 145, 146, 386–388],

▪ 92 intervention studies where PARIHS did not guide the development of an intervention [149, 152, 153, 155, 156, 158, 160, 162, 167, 168, 171, 176, 178, 179, 182–185, 194, 201–203, 205–209, 211, 212, 214, 217, 219–223, 225, 234–236, 243–245, 249–252, 254, 255, 258–261, 263, 265, 266, 268–270, 273, 274, 276–285, 287–292, 296, 297, 299–301, 303–312],

▪ 72 non-intervention studies [150, 151, 154, 157, 159, 161, 163–166, 169, 170, 172–175, 177, 180, 181, 186–193, 195–200, 204, 210, 213, 215, 216, 218, 224, 226–233, 237–242, 246–248, 253, 256, 257, 262, 264, 267, 271, 272, 275, 286, 293–295, 298, 302]

In addition, the database included 28 empirical review studies [3, 13, 313, 314, 316–338, 389] and 49 opinion/theoretical articles [2, 144, 339–385]. In terms of professional focus, about 65% of the included articles involved nursing.

In the following sections, references have been added to the categorical items in the data extraction while we have opted only to provide examples of references to the findings from the qualitative exploration of how the PARIHS framework was operationalized in detail.

### Settings

Of the articles reporting type of setting where the implementation project/research took place, a majority were undertaken in hospitals (*n* = 126) [26, 27, 30, 38, 39, 41, 42, 45, 49, 51–55, 60, 62–64, 67, 70–72, 77, 84, 85, 88, 89, 95–98, 103, 106, 107, 111, 115–118, 120–122, 128, 131, 132, 136, 137, 139, 143, 146, 149, 151, 153–155, 157, 161, 165, 167–169, 172, 173, 175, 180–182, 184, 186, 190–193, 205, 207, 212, 215, 221, 223, 227, 230, 231, 240, 243, 244, 246, 248–251, 256, 258, 260, 265, 268, 271, 273–275, 277–279, 281, 282, 284, 288–290, 294, 296, 298, 299, 301, 305, 306, 312, 314, 320, 336, 352, 353, 360, 362, 382, 386, 388], followed by a combination of multiple healthcare settings (*n* = 80) [31, 35, 37, 47, 50, 56, 65, 66, 68, 73, 79, 92, 99, 100, 109, 110, 112, 114, 133, 138, 144, 145, 150, 159, 160, 162, 163, 171, 177, 183, 187, 194, 197, 204, 208–210, 216, 218, 219, 226, 228, 233, 238, 245, 253, 262, 264, 276, 280, 283, 287, 292, 293, 295, 302, 307–310, 313, 321, 323, 326, 327, 329, 330, 333, 337, 338, 344, 345, 365, 367, 368, 371, 373, 376, 387, 389], community/social care settings (*n* = 54) [24, 34, 36, 43, 44, 46, 48, 57, 59, 61, 69, 74–76, 80, 81, 86, 87, 94, 102, 104, 108, 113, 123, 129, 134, 142, 152, 156, 158, 174, 178, 185, 195, 201, 203, 214, 217, 222, 229, 232, 241, 257, 263, 266, 269, 270, 300, 304, 351, 355, 357, 363], primary health care (*n* = 34) [23, 25, 28, 29, 40, 58, 82, 90, 93, 119, 125, 130, 140, 141, 164, 170, 196, 198, 200, 202, 224, 225, 235, 236, 242, 252, 259, 261, 272, 285, 286, 291, 332, 340], and home-based care (*n* = 7) [78, 91, 105, 166, 254, 255, 311]. Five articles were derived from special settings such as construction [176], education [101], pharmacies [135], urban planning [383], and public health institutions [32]. In 44 articles [2, 3, 13, 33, 124, 126, 127, 179, 206, 211, 220, 237, 239, 247, 297, 316, 317, 322, 324, 325, 328, 339, 341–343, 346–350, 354, 356, 358, 364, 369, 372, 374, 375, 377, 378, 380, 381, 384, 385], the setting was not reported or not applicable (e.g., opinion/theoretical articles). For empirical studies and published protocols, about 28% were derived from research in the USA, 22% from Canada, 10% from Sweden, and 10% from the UK. The remaining articles mainly originated from other high-income countries in Europe; in addition, there were a few articles reporting studies in low- and middle-income countries, including Vietnam, Tanzania, Mozambique, and Uganda [46, 82, 110, 150, 235, 287].

### Timing of different types of articles

**Fig. 2:**
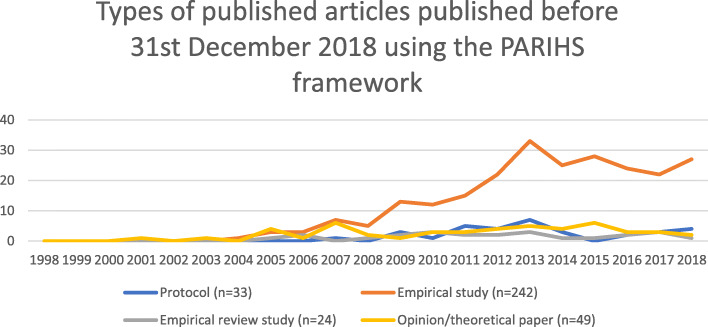
Types of articles published before December 31 using the PARIHS framework

The types of articles published using the PARIHS framework changed over time, with an increase in the number of empirical studies from 2004 onwards, as illustrated in Fig. 2. As the search for articles for this review only included the first eight months of 2019, the graph is limited to full years (i.e., 1998 through 2018).

### Use of PARIHS

**Fig. 3:**
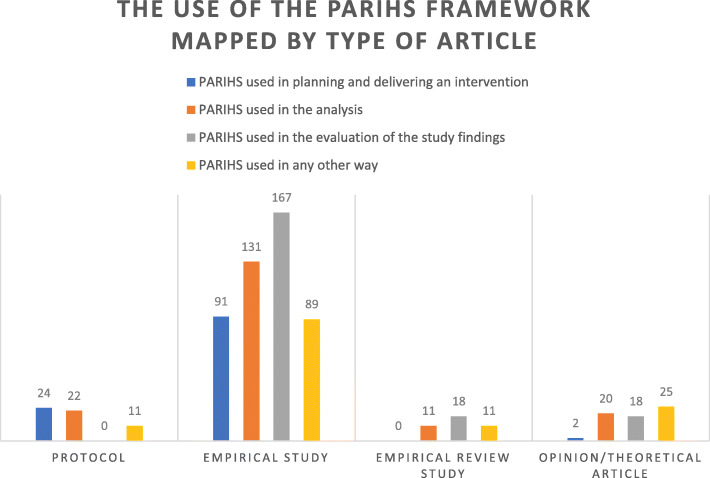
Use of the PARIHS framework by type of article

Figure 3 depicts how PARIHS was used by type of article. Although authors frequently claimed that PARIHS was used in one or more ways, details as to how the framework was used were often lacking.

#### The application of PARIHS to plan and deliver an intervention

In total, 117 (32%) articles claimed to use the PARIHS *framework to plan and deliver an intervention* [23–46, 58–82, 84–143, 145, 146, 339, 340, 386–388]. Predominantly, these were empirical studies (*n* = 91) [58–82, 84–143, 145, 146, 386–388] but also two opinion/theoretical articles [339, 340] and 24 protocols [23–46]. Of the 117 articles, about half stated that the framework was used for theoretically informing, framing, or guiding an intervention (e.g., [82, 103, 105, 134, 386]). However, in these studies, PARIHS was referred to only in a general sense, in that the core elements of the framework were said to have informed the planning of the study. There was a lack of detail provided about what elements of the framework were used and how they were operationalized to plan and deliver an intervention. In the other half of the 117 articles, it was described more specifically that one or more elements of the framework had been used. Most commonly the facilitation element (e.g., [58, 80, 92, 98, 110]) was referred to as guiding an implementation strategy. The articles that provided explicit descriptions of interventions using facilitation employed strategies such as education, reminders, audit-and-feedback, action learning, and evidence-based quality improvement, and roles including internal and external facilitators and improvement teams to enable the uptake of evidence (e.g., [23, 79, 125, 142]). Some articles drew on the PARIHS framework more specifically, to understand the role of organizational context in implementation (e.g., [34, 63, 145, 340]).

#### The application of PARIHS in data analysis

There were 184 (50%) articles where the PARIHS framework was reported to be used in the analysis [2, 13, 23–35, 47–55, 58–82, 84–94, 149–242, 313, 314, 316–322, 339, 341–358, 386, 389]. Most of these involved empirical studies (*n* = 131) [58–82, 84–94, 149–242, 386] where PARIHS often was described as guiding or framing the data collection, e.g., developing an interview guide, and/or analysis, but with no further details. In articles that provided more detailed information, PARIHS was used to guide or frame qualitative analyses in about 50 studies (e.g., [67, 94, 173, 178, 207]). Of these, around 20 used a deductive approach in that they used the elements and sub-elements to structure the analytic process (e.g., [150, 170, 188, 215, 242]). About 35 studies applied PARIHS for quantitative analysis, (e.g., [69, 168, 174, 190, 211]). In half of these, the Alberta Context Tool (e.g., [155, 165, 180, 195, 229]) and the Organizational Readiness to Change Assessment Tool (e.g., [74, 159, 219, 240]) were used; both these tools being derived from PARIHS. Empirical studies using the PARIHS framework in the analysis encompassed primarily all three main elements of PARIHS (e.g., [166, 181, 193, 221]) and the context domain (e.g., [78, 152, 153, 207]), and in lesser extent the evidence (e.g., [185, 208, 214]) and the facilitation domain (e.g., [58, 67, 79, 182]).

Eleven review studies [13, 313, 314, 316–322, 389] used the framework for the analysis; findings were mapped to PARIHS elements in a few studies [316, 317, 389]; one described that their data had been “analysed through the lens of PARIHS” (p1) [389]. A couple of the review studies had PARIHS as the object for analysis, comparing it with other frameworks [318, 322]. This approach was also common in the 20 opinion/theoretical articles [2, 339, 341–348, 350–358], where the PARIHS framework itself was the focus of the analysis (e.g., [341, 349, 354]). In these articles, the analysis was performed in different ways, primarily through mapping and comparing PARIHS to other frameworks or models or even policies, but also for general discussions on implementation and evidence-based practice. Among the 185 articles that reported using the PARIHS framework in the analysis, there were also 22 protocols where authors reported that the intention was to use the framework in the analysis [23–35, 47–55].

#### The application of PARIHS in the evaluation of study findings

A total of 203 (55%) included articles provided information on how the PARIHS framework was used in the evaluation of study findings, in terms of contributing to the discussion and interpretation of results [13, 52, 58–82, 84–89, 95–121, 149–214, 243–259, 261–284, 313, 314, 316–320, 323–331, 339, 341–350, 359–365, 386, 389]. The majority (*n* = 167) of these were empirical studies [52, 58–82, 84–89, 95–121, 149–214, 243–259, 261–284, 386].

We found two main approaches to how the PARIHS framework was used in the evaluation of study findings. First, PARIHS was used to organize the discussion of the findings (e.g., [73, 87, 109, 166, 214]), where the framework and/or its elements were used to provide a structure for reporting or generally discussing the findings, or both, for example in stating that the key elements of PARIHS were reflected in the study findings. Second, the framework was used to consider the implications of the study’s findings (e.g., [81, 84, 170, 207, 361]), where the framework or its elements (varying between one (e.g., [75, 195, 211]), two (e.g., [71, 86, 105]), and all the three main elements (e.g., [80, 261, 269])) enabled authors to elaborate on findings, or reflect on the implications of their study to evaluate the PARIHS framework itself. Specifically, we found some empirical articles reported evaluating the PARIHS element “context” by means of context tools (e.g., [155]). In addition, an evaluation of the study findings using the framework was identified in 18 opinion/theoretical articles [339, 341–350, 359–365] and 18 empirical review studies [13, 313, 314, 316–320, 323–331, 389]. Among the opinion/theoretical articles, there were papers evaluating other theoretical constructions in relation to the PARIHS framework (e.g., [364]).

#### The application of PARIHS in any other way

A total of 136 (37%) reported using PARIHS in some other way than directly informing the planning and delivery of an intervention or analyzing and evaluating findings [3, 23–25, 36, 37, 47–50, 56–62, 90, 95–97, 122–127, 144, 149–170, 215–224, 243–256, 285–313, 323, 324, 332–338, 351, 359–361, 366–385]. A majority of these articles (*n* = 89) were empirical studies [58–62, 90, 95–97, 122–127, 149–170, 215–224, 243–256, 285–312], and about half of these described the use of PARIHS as an overall guide to frame the study (e.g., [58, 60, 168, 222, 285, 303]). A similar finding was apparent in the 11 protocols [23–25, 36, 37, 47–50, 56, 57]; about half of these also referred to the use of PARIHS to guide and frame the study design (e.g., [47, 48, 50, 57]).

An alternative use of PARIHS in empirical studies involved focusing on one of the three PARIHS elements (*n* = 17) and investigating them in greater depth, most notably context (*n* = 10) (e.g., [155, 232]) and facilitation (*n* = 7) (e.g., [307, 312]). A total of 25 opinion/theoretical articles [144, 351, 359–361, 366–385] reported using the PARIHS framework in some other way, including a discussion about PARIHS as part of presenting a general overview of theories and frameworks to inform implementation (e.g., [369, 376, 378, 384]), using PARIHS to augment, develop, or evaluate other implementation models and frameworks (e.g., [318, 359, 367, 374, 382]), and informing education and learning and teaching initiatives [144, 372]. Empirical review articles (*n* = 11) included reviews of implementation frameworks [3, 313, 323, 324, 332–338], including PARIHS, a review of the facilitation dimension of PARIHS and a discussion of the potential to combine implementation and improvement methodologies.

#### Testing and providing views on the validity of the framework

A total of 102 (28%) articles described testing or validating PARIHS, or provided comments on the validity of the framework [3, 13, 23, 24, 35, 44, 46, 58, 60, 62, 64, 67, 71, 74, 76, 78–81, 84, 85, 89, 98, 105, 107, 110, 113, 115, 120, 121, 143, 149, 150, 153, 155, 157–159, 166, 168, 170, 172, 180–182, 187, 188, 190, 191, 195, 198, 201, 203, 204, 206–209, 211, 212, 214, 229, 246, 249, 250, 252, 253, 255, 264, 267, 268, 277, 278, 280, 281, 287, 303, 308, 314, 316–319, 322, 323, 326, 330, 332, 333, 335, 336, 341, 342, 345–347, 349, 359, 364, 369, 381, 386]. Of these, 72 were empirical studies [4, 58, 60, 62, 64, 67, 71, 74, 76, 78–81, 84, 85, 89, 98, 105, 107, 110, 113, 115, 120, 121, 143, 149, 150, 153, 155, 157–159, 166, 168, 170, 172, 180–182, 187, 188, 190, 191, 195, 198, 201, 203, 204, 206–209, 211, 212, 214, 229, 246, 249, 250, 252, 253, 255, 264, 267, 268, 277, 278, 280, 281, 287, 303, 308, 386], five were study protocols [23, 24, 35, 44, 46], 10 opinion/theoretical articles [341, 342, 345–347, 349, 359, 364, 369, 381], and 15 empirical reviews [3, 13, 314, 316–319, 322, 323, 326, 330, 332, 333, 335, 336]. Empirical studies either tested the whole or parts of the framework with a focus on:

▪ The validity of the whole framework (e.g., [24, 74, 157, 195, 209])

▪ The validity of context (e.g., [155, 190, 280, 287, 308])

▪ The validity of facilitation (e.g., [23, 58, 182, 206])

▪ The validity of evidence (e.g., [255])

▪ Identifying gaps in the framework (e.g., [64, 170, 326])

Over the review study period (1998 to 2019), among empirical studies, there was a shift from primarily studying the context element of the framework to more articles evaluating the whole framework. This was also evident in the pattern found in the protocols, which mostly focused on testing facilitation (e.g., [58, 182, 206]). Opinion/theoretical articles tended to critique the whole framework (e.g., [319, 326, 342, 349, 369]). Of the 15 empirical reviews, the majority focused on the whole framework (e.g., [13, 322, 333]), then on context (e.g., [316, 318, 335]) and then on facilitation (e.g., [323]). Of note is the lack of attention in the literature to the element of “evidence” in the PARIHS framework (examples of articles paying attention to evidence include [208, 255]).

The articles varied in detail, depth, and quality in terms of descriptions of how they went about testing the validity of the PARIHS framework. Approaches ranged from general observations of whether the research teams/users found the elements and sub-elements easy to use (e.g., [62, 188, 203]), to studies that used elements of context described in the PARIHS framework to validate new context measures across settings and groups (e.g., [150, 155, 207]). As one example, the Alberta Context Tool started from the PARIHS conceptualization of context to include dimensions of culture, leadership, and evaluation.

Regarding the strength and limitations of the PARIHS framework, about one third of the included articles reported on its strengths and about 10% commented on perceived limitations. The identified strengths included:

▪ Holistic implementation framework (e.g., [141, 164, 209, 258]) that is perceived as intuitive and accessible.

▪ Both practical and theoretical and therefore feasible to use by both clinicians and researchers; also seen as intuitive to use and accessible (e.g., [117, 209, 255]).

▪ Can be used as a tool: diagnostic/process/evaluative tool; predictive/explanatory tool or as a way to explain the interplay of factors (e.g., [93, 205, 255, 285, 379]).

▪ Can accommodate a range of other theoretical perspectives (including approaches such as social network theory, participatory action research, coaching, change management and other knowledge translation frameworks) (e.g., [93, 105, 245, 246]).

▪ Can be used successfully in a range of different contexts (low- and middle-income countries) [150] and services and for various groups of patients (disability, aged care) (e.g., [80, 113, 248, 312]).

Limitations of the PARIHS framework included:

▪ Poor operationalization of key terms leading to difficulties in understanding and an overlap of elements and sub-elements (e.g., [165, 285, 376]).

▪ Lack of practical guidance on steps to operationalize the framework (e.g., [209, 254]) with a subsequent lack of tools.

▪ Lack of information on the individual and their characteristics (e.g., [209, 361]) and their lack of understanding of evidence (e.g., [204, 390]).

▪ Too structured and does not acknowledge the multidimensionality and uncertainty of implementation (e.g., [143, 214]).

▪ Lack of acknowledgement of wider contextual issues such as the impact of professional, socio-political, and policy issues on implementation (e.g., [115, 143, 285, 354]).

▪ Not providing support in how to overcome barriers to successful implementation (e.g., [88]).

## Discussion

In a recent survey among implementation scientists, the PARIHS framework was found to be one of the sixth most commonly used theoretical frameworks [4]. Yet, in our review, about 23% (*n* = 367) of the identified 1614 articles citing any of the four selected core PARIHS articles used the framework in any substantial way. Similarly, a review of the CFIR found that 26/429 (6%) of articles citing the framework were judged to use the framework in a meaningful way (i.e., used the CFIR to guide data collection, measurement, coding, analysis, and/or reporting) [12]. A citation analysis of the KTA framework found that about 14% (146/1057) of screened abstracts described using the KTA to varying degrees, although only 10 articles were judged to have applied the framework in a fully integrated way to inform the design, delivery, and evaluation of implementation activities [11].

PARIHS has been used in a diverse range of settings but, similarly to other commonly used implementation frameworks, most often superficially or partially. The whole framework has seldom been used holistically to guide all aspects of implementation studies. Implementation science scholars have repeatedly argued that the underuse, superficial use, and misuse of implementation frameworks might reduce the potential scientific advancements in the field, but also the capacity for changing healthcare practice and outcomes [4]. The rationale for not using the whole PARIHS framework could be many, including the justified reason of only being interested in a particular element. As such, partial use cannot always be considered as inappropriate. Simultaneously, many researchers entering the field might be overwhelmed with the many frameworks available and the lack of guidance about how to select and operationalize them and using their elements [2, 4, 391]. The current citation analysis can thus help remedy a gap in the literature by revealing how the PARIHS framework has been used to date, in full or partially, and thus provides input to users of its potential use.

The use of theoretical frameworks in implementation science serves the purpose of guiding researchers’ and practitioners’ implementation plans and informing their approaches to implementation and evaluation. This includes decisions about what data to gather to describe and explain implementation, their hypotheses about action steps needed, how to account for the critical role of context, and providing a foundation for analysis and discussion [7]. The advancement of theoretically informed implementation science will, however, depend on much improved descriptions as to why and how a certain framework was used, and an enhanced and better-informed critical reflection of the functionality of that framework. This review shows that the PARIHS framework has rarely been used as a whole; rather, certain elements tend to be applied, often retrospectively as indicated in Fig. 3 underlining the use of PARIHS in the evaluation of study findings, which resonates with the findings of reviews about the use of the KTA [2] and CFIR [11] frameworks. This could be as a result of a lack of theoretical coherence of some frameworks making them difficult to apply holistically, and/or a function of a general challenge that researchers face in operationalizing theory. However, this could also be a result of publishing constraints. While the PARIHS framework may have guided implementation or been implicitly used in the study design, it was rarely the focus of the publications. Further, the aims and scopes of scientific health care journals have historically prioritized clinical outcomes over implementation outcomes where one could expect a more detailed description of the use of theoretical frameworks. This may have resulted in authors not fully reporting their use of, e.g., the PARIHS framework.

The number of empirical studies using the PARIHS framework has steadily increased over the review period. There is also evidence to show that more research teams have contributed to critiquing the framework in terms of reporting on its strengths and limitations and its validity. The pattern of investigation is moving from studies on context, to more systematic explorations of facilitation, thus contributing to a more detailed understanding of the elements and sub-elements of the framework. The lack of focus on “evidence” identified in this review highlights the need for researchers and clinicians to focus on the multi-dimensionality of what is being implemented. Common patterns emerging in this review support the changes made to the most recent refinement of the PARIHS framework [359].

Consistent with other reviews of the use of theoretical frameworks in implementation science, we found that PARIHS was often not used as intended. Further, it was not always clear why the particular framework was chosen. Frequently, authors merely cite a framework without providing any further information about how the framework was used. The lack of clear guidance on how to operationalize frameworks might be one of the underlying reasons for this. Lastly, to enable a critical review of frameworks and further build collective understanding of implementation, we urge authors to be more explicit about how theory informs studies. Development and adoption of reporting guidelines on how framework(s) are used in implementation studies might assist in sharpening the link between the used framework(s) and the individual study, but could potentially also enhance the opportunities for advancing the scientific understanding of implementation.

### Limitations

To increase study reliability during the review process, more than one person identified, assessed, and interpreted the data. We had regular meetings to discuss potential difficulties in assessing included articles, and subsequently, all decisions were resolved by consensus to enhance rigor. We used a rigorous search strategy, which was undertaken by an information specialist. The standardization of our processes across the team was also enhanced by the creation of an online data extraction form via Google. However, as the form was not linked to other software (e.g., Endnote), this added time-consuming processes.

As we did not include articles that were not written in English, we may have limited the insights about the application of the PARIHS framework, particularly with relevance to different country contexts. Additionally, we did not search the grey literature for practical reasons concerning the size of the literature, which may also have provided some additional insights not reflected in this publication. We also limited our search to two databases, which may mean we missed some relevant articles. However, we are confident that we found the majority of relevant published evidence to address the review questions because of a rigorous approach to retrieval. Thus, we think the findings of our citation analysis on the use of PARIHS are generalizable for studies in English published in peer-reviewed journals.

## Conclusions

The importance of theoretically underpinned implementation science has been consistently highlighted. Theory is important for maximizing the chances of study transferability, providing an explanation of implementation processes, developing and tailoring implementation interventions, evaluating implementation, and explaining outcomes. This review of the use of the PARIHS framework, one of the most cited implementation frameworks, shows that its actual use and application has been frequently partial and generally not well described. Our ability to advance the science of implementation and ultimately affect outcomes will, in part, be dependent on better use of theory. Therefore, it is incumbent on theory developers to generate accessible and applicable theories, frameworks, and models, and for theory users to operationalize these in a considered and transparent way. We propose that the development and adoption of reporting guidelines on how framework(s) are used in implementation studies might enhance the maturity of implementation science.

## Supplementary information

**Additional file 1:** Form for initial assessment and form for data extraction

## Data Availability

The datasets generated and analyzed during the current study can be obtained through contacting the first author.
