# Supplementary material for: The use of the PARIHS framework in implementation research and practice—a citation analysis of the literature
Source: Implement Sci. 2020 Aug 27;15:68. doi: 10.1186/s13012-020-01003-0 (PMC7450685; doi:10.1186/s13012-020-01003-0)
Supplement: Supplementary file 1 — Additional file 1: Form for initial assessment and form for data extraction [file 13012_2020_1003_MOESM1_ESM.pdf]

Initial assessment for PARIHS citation analysis

* Required

Assessment question	Answer option(s)
1. First author? *	*Free text*
2. Year of publication? *	1998 □<br>1999 □<br>2000 □<br>2001 □<br>2002 □<br>2003 □<br>2004 □<br>2005 □<br>2006 □<br>2007 □<br>2008 □<br>2009 □<br>2010 □<br>2011 □<br>2012 □<br>2013 □<br>2014 □<br>2015 □<br>2016 □<br>2017 □<br>2018 □
3. Full title of reviewed publication? *	*Free text*
4. Does the reviewed publication cite *Kitson (1998), Enabling the implementation of evidence-based practice: a conceptual framework*? *	Yes □ No □
5. Does the reviewed publication cite *Kitson (2008), Evaluating the successful implementation of evidence into practice using the PARiHS framework: theoretical and practical challenges*? *	Yes □ No □
6. Does the reviewed publication cite *Rycroft-Malone (2002), Ingredients for change: revisiting a conceptual framework*? *	Yes □ No □
7. Does the reviewed publication cite *Rycroft-Malone (2004) An exploration of the factors that influence the implementation of evidence into practice*? *	Yes □ No □
8. Is the publication a peer-reviewed scientific article? *	Yes □ No □
9. Does the article do more than cite the PARIHS framework? *<br><br>This question determines if the article will be used for full data extraction, i.e. PARIHS used in planning, analyzing and/or evaluating a study (or theoretical article).	Yes □ No □

Data extraction for PARIHS citation analysis

Please read the instruction to all the items and always leave comment when answering *Yes* to the open-ended questions. Copy-paste from the article when referring and add information on what page of the article that the information was captured from. It is key that we do not 'value the quality' of the study at this stage but that the assessment is made based on what is written in the article.

* Required

Assessment question	Answer option(s)
1. Who are you? *	Alison ☐<br>Ankie ☐<br>Anna B ☐<br>Anna E ☐<br>Gill ☐<br>Ian ☐<br>Jo ☐<br>Lars ☐
2. Article ID? *	*Free text*
3. What type of article is it? *	Empirical study ☐<br>Empirical review study ☐<br>Opinion/theoretical paper ☐<br>Protocol ☐<br>Other: ______________ ☐
4. Was the PARIHS used in planning and delivering an intervention? *<br><br>NB: Intervention/implementation informed by PARIHS. The option N/A applies when there is no intervention/implementation. 'Intervention' should be seen as both including research intervention and 'practice development/improvement'. NB: Choose 'Yes' for Protocol where PARIHS is stated to be used in planning for an intervention and provide information in Q5.	Yes ☐ No ☐ N/A ☐
5. If YES to the above, how?	*Free text*
6. Was the PARIHS used in the analysis? *<br><br>e.g. making sense of data. Framing the data according to PARIHS. Also including theoretical articles. NB: Choose 'Yes' for Protocol where PARIHS is stated to be used in the analysis and provide information in Q7.	Yes ☐ No ☐ Protocol, *PARIHS planned to be used in analysis* ☐
7. If YES to the above, how?	*Free text*
8. Was the PARIHS used in the discussion of the study findings? *<br><br>NB: Understanding/interpretation/discussion. Also including theoretical articles. NB. The N/A option applies only to Protocols.	Yes ☐ No ☐ N/A ☐
9. If YES to the above, how?<br><br>Make a distinction if PARIHS is just cited in the discussion or if PARIHS is used to evaluate the findings.	*Free text*
10. Was the PARIHS used in any other way than 4, 6, 8?	Yes ☐ No ☐

e.g. mentions that PARIHS framed the study but provides no or little details on how. NB: Choose 'Yes' for Protocol where PARIHS is mentioned to be used in any other way than Q4 and Q6 and provide information in Q11.	
11. If YES to the above, how?<br><br>e.g.: Providing rationale for the study, Guiding framework for the study, Informing the framework that guided the study, Informing tool development/data collection, Informing understanding of PARIHS elements, Influencing the authors thinking on study design.	*Free text*
12. Is the article testing/validating or providing some commentary on the validity of the PARIHS?<br><br>NB: 'Yes' applies even if only one/two of the constructs of PARIHS are studied/validated.	Yes ☐ No ☐
13. If YES to the above, how?<br><br>NB: State if the paper is explicitly testing or validating the framework or if the findings provide comments on the validity of parts of or the whole PARIHS.	*Free text*
14. Countrie(s) - only if applicable<br><br>NB: The question is primarily asking about where the work was carried out (rather than where authors come from). If several countries please separate by comma (this also applies to reviews where the included studies might derive from several countries). If not reported please write: Not reported	*Free text*
15. Does the article involve nursing?<br><br>NB: Midwifery is NOT considered nursing in this case. Whether nurses were involved should be explicitly stated in the paper and not assumed, if 'Yes' should be ticked off	Yes ☐ No ☐
16. If other/more than nursing (15) - which disciplines and roles are involved?	*Free text*
17. What setting does the publication derive from?<br><br>NB: It is possible to tick several options. Please note that Hospital includes primary, secondary and tertiary hospitals. Nursing homes should be included under 'Community/Social care', If other settings make a note under Q19. Some publications might not fit under these options - please use 'Not reported' and make a comment at Q19.	Hospital setting ☐<br>Primary health care setting ☐<br>Home-based care ☐<br>Community/Social care setting ☐<br>Multiple settings ☐<br>Not reported ☐
18. What do authors report as the strengths and limitations of the PARIHS?<br><br>NB: Leave empty if strengths/limitations are not mentioned.	*Free text*
19. Additional comments<br><br>NB1: Especially think about things that might give some flesh to the bone to discussing our findings. NB2: For opinion/theoretical papers and reviews note if the paper is supportive, critical (pointing at deficiencies) or expanding on PARIHS.	*Free text*
